# ANXA1 improves mitochondrial homeostasis through uncoupling protein 1 in diabetic nephropathy

**DOI:** 10.1016/j.jare.2025.11.039

**Published:** 2025-11-22

**Authors:** Zi-Han Li, Lu Fang, Liang Wu, Dong-Yuan Chang, Manyuan Dong, Liang Ji, Qi Zhang, Ming-Hui Zhao, Sydney C.W. Tang, Lemin Zheng, Min Chen

**Affiliations:** a Renal Division, Department of Medicine, Peking University First Hospital, Peking University Institute of Nephrology, Key Laboratory of Renal Disease, Ministry of Health of China, Key Laboratory of Chronic Kidney Disease Prevention and Treatment (Peking University), Ministry of Education, Research Units of Diagnosis and Treatment of Immune-mediated Kidney Diseases, Chinese Academy of Medical Sciences, China; b Department of Nephrology, Peking University First Hospital, State Key Laboratory of Vascular Homeostasis and Remodeling, Peking University, Beijing, China; c Research Units of Diagnosis and Treatment of Immune-mediated Kidney Diseases, Chinese Academy of Medical Sciences, China; d Peking-Tsinghua Center for Life Sciences, Beijing 100034, China; e The Institute of Cardiovascular Sciences and Institute of Systems Biomedicine, School of Basic Medical Sciences, State Key Laboratory of Vascular Homeostasis and Remodeling, NHC Key Laboratory of Cardiovascular Molecular Biology and Regulatory Peptides, Beijing Key Laboratory of Cardiovascular Receptors Research, Health Science Center, Peking University, Beijing 100191, China; f Beijing Tiantan Hospital, China National Clinical Research Center for Neurological Diseases, Advanced Innovation Center for Human Brain Protection, Capital Medical University, Tiantan Xili, Chongwen District, Beijing 100050, China; g Division of Nephrology, Department of Medicine, School of Clinical Medicine, The University of Hong Kong, Queen Mary Hospital, Hong Kong, China

**Keywords:** Diabetic nephropathy, Uncoupling protein 1, Annexin A1, Mitochondrial dynamics, GATA binding protein 3, Cardiolipin synthase 1

## Abstract

**Introduction:**

Diabetic nephropathy (DN) is a major public health concern. Our previous study found that annexin A1 (ANXA1) alleviated DN by improving mitochondrial homeostasis. However, the underlying mechanism is not fully clear yet.

**Objectives:**

This study aimed to explore mechanisms by which ANXA1 improves mitochondrial homeostasis and identify novel therapeutic targets for DN.

**Methods:**

Diabetic *Anxa1^-/-^*/uncoupling protein 1 (*Ucp1*)*^-/-^* mice and renal *Ucp1*/cardiolipin synthase 1 (*Crls1*)-overexpressing mice were constructed. Diabetes was established using high-fat diet (HFD) plus streptozotocin (STZ). CL316243 was used to upregulate UCP1 in *db/db* mice. Knockdown and overexpression in proximal tubular epithelial cells (PTECs) were constructed. Metabolomics and lipidomics were applied.

**Results:**

Transcriptomics revealed that *Ucp1* was the most significantly downregulated mitochondria-associated gene in kidneys of diabetic *Anxa1*-KO mice. Functional validation demonstrated that local overexpression of *Ucp1* in kidneys alleviated urine albumin-to-creatine ratio (uACR), kidney injuries in histopathology and mitochondrial fission in *Anxa1*-KO diabetic mice. *In vitro*, *UCP1* silencing abolished the improvements of human recombinant ANXA1 on high glucose (HG)-induced inflammation, fibrosis and mitochondrial dysfunction in PTECs. Silencing/overexpression of *ANXA1* reduced/increased the stability of transcription factor GATA binding protein 3 (GATA3) in HK-2 cells under HG conditions, respectively. GATA3 could bind to the proximal promoter region of *UCP1*, thereby enhancing its transcriptional activity. UCP1 was significantly upregulated in kidneys of DN patients and mice. UCP1 deficiency reduced renal cardiolipin and exacerbated diabetes-induced kidney injury, including aggravated uACR, interstitial inflammation and fibrosis, and enhanced mitochondrial fission. Mechanistically, UCP1 upregulated CRLS1 by modulating aristaless-related homeobox (ARX), thereby promoting cardiolipin biosynthesis and reducing mitochondrial fission. Therapeutically, pharmacological upregulation of UCP1 by CL316243 attenuated established DN in *db*/*db* mice.

**Conclusion:**

ANXA1 stabilized GATA3 to upregulate UCP1, which promoted cardiolipin biosynthesis through the ARX/CRLS1 axis, inhibited mitochondrial fission, thereby alleviated DN. This study highlighted the therapeutic potential of targeting UCP1 in DN.

## Highlights

• Annexin A1 mitigated DN progression by enhancing UCP1-mediated mitochondrial homeostasis.
• Annexin A1 increased UCP1 expression by stabilizing the transcription factor GATA3.
• UCP1 maintained mitochondrial morphology and reduced mitochondrial fission in the progression of DN.
• UCP1 promoted cardiolipin synthesis through the ARX/CRLS1 pathway and suppressed mitochondrial fission.
• Pharmacological UCP1 activation with CL316243 effectively attenuated DN in *db/db* mice.

## Graphical abstract

**Figure:**
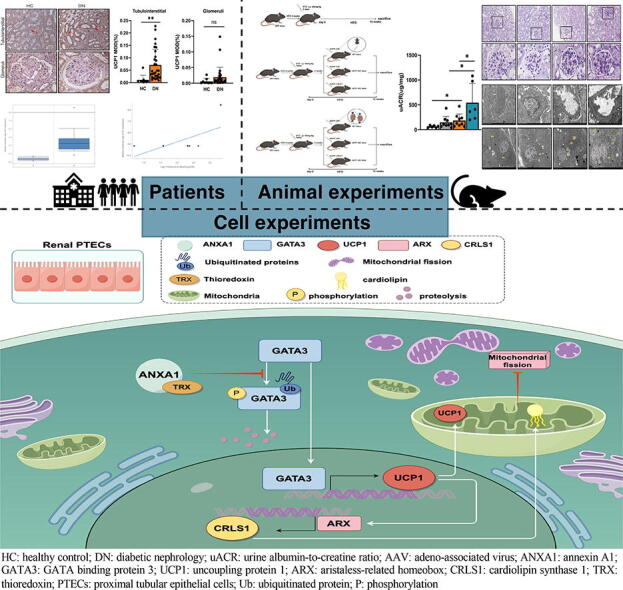

## Introduction

Diabetic nephropathy (DN), a major complication of diabetes mellitus (DM), is clinically characterized by increased urinary albumin excretion and/or decreased glomerular filtration rate (GFR) [1]. It accounts for a substantial proportion of chronic kidney disease (CKD) and remains the leading cause of end-stage renal disease (ESRD) worldwide [2,3]. The pathogenesis of DN is highly complex and remains not fully clear yet. Chronic hyperglycemia increases mitochondrial reactive oxygen species (ROS) production through excessive electron leakage in the respiratory chain, glucose autoxidation, protein kinase C activation, and the hexosamine and polyol pathways [4]. Meanwhile, persistent hyperglycemia promotes the accumulation of advanced glycation end-products (AGEs), which activate the receptor for AGEs (RAGE) signaling pathway, further amplify oxidative stress, inflammation and fibrosis [5]. These processes contribute to glomerulosclerosis, tubular atrophy and interstitial fibrosis, which are histological characteristics of advanced DN.

Given the central role of oxidative stress and inflammation in DN, the process of promoting inflammation resolution has gained increasing attention. Annexin A1 (ANXA1), a 37kDa calcium-dependent phospholipid-binding protein, is a typical pro-inflammatory resolving protein [6]. Previous studies demonstrated that ANXA1 exerted protective effects in diabetic complications. In a murine model of type 1 diabetes, ANXA1 attenuated microvascular complications by restoring protein kinase B (Akt) signaling, thereby improving both cardiac and renal outcomes. In type 2 diabetes, ANXA1 was identified as an endogenous regulator of Ras homolog family member A (RhoA), and its activation ameliorated metabolic dysfunction and insulin resistance, underscoring its therapeutic potential in metabolic diseases [7,8]. In cancer, ANXA1 was shown to influence mitochondrial function by modulating apoptosis and autophagy pathways [9]. Furthermore, ANXA1 was found to protect against the development of heart failure by regulating blood pressure and maintaining mitochondrial function, with sex-specific difference observed in its cardioprotective effects [10]. These findings collectively supported the notion that ANXA1 played a central role in mitochondrial regulation and tissue protection under metabolic stress. Our recent studies found that ANXA1 alleviated DN by improving mitochondrial morphology and function [11,12]. However, the precise mechanism by which ANXA1 regulated mitochondrial homeostasis in DN is not fully clear yet. Given the importance of mitochondrial homeostasis in DN, some previous studies also explored various therapeutic approaches targeting mitochondrial function. Antioxidants, such as N-acetylcysteine and nicotinamide adenine dinucleotide-dependent deacetylase sirtuin 1 (SIRT1) activator, showed renal protective effects by reducing mitochondrial oxidative stress [13,14]. Thus, this study aimed to investigate the molecular mechanisms by which ANXA1 modulates mitochondrial homeostasis, and to find clues for the therapeutic potential of mitochondrial targeted treatment in DN.

Further analysis of kidney tissues from our previous study [12] revealed, via transcriptomic profiling, that uncoupling protein 1 (UCP1, encoded by *Ucp1*) was the most significantly downregulated gene among those related to fatty acid oxidation (FAO) and mitochondrial function in the renal cortex of diabetic *Anxa1*-KO mice compared to diabetic wild-type (WT) mice. As an important component and the most abundant member in the uncoupling protein family, UCP1 mediates oxidative phosphorylation and uncoupling [15]. Upon activation, UCP1 facilitates proton leak across the inner mitochondrial membrane, dissipating the proton gradient without adenosine triphosphate (ATP) production and thereby reducing mitochondrial ROS generation [16]. Thus, UCP1 plays a role in oxidative stress-related diseases and metabolic syndromes [15,16]. UCP1, which is downregulated during acute kidney injury due to suppression of the peroxisome proliferator-activated receptor gamma (PPARγ)/UCP1 pathway, plays a key role in protecting against cellular death [17] and limits injury progression by reducing lipid accumulation [18]. Also, its activation could alleviate fibrosis and delay DN progression [19]. These findings suggested a potential link between ANXA1 and UCP1 in mitochondrial regulation, with therapeutic implications of targeting UCP1 in kidney diseases. Therefore, we hypothesized that ANXA1 improved mitochondrial homeostasis in DN by regulating UCP1.

In this study, it was found that ANXA1 improved mitochondrial homeostasis by upregulating UCP1 in DN. Specifically, *Ucp1* overexpression substantially rescued kidney injuries, including urine albumin-to-creatine ratio (uACR), renal pathology and mitochondrial fission, in diabetic *Anxa1*-KO mice. Moreover, in HK-2 cells and primary murine proximal tubular epithelial cells (PTECs), *UCP1* silencing abolished the protective effects of human recombinant ANXA1 (hrANXA1) against high glucose (HG)-induced inflammation, fibrosis, and mitochondrial dysfunction. Mechanistically, ANXA1 reduced the hydrolysis of transcription factor GATA binding protein 3 (GATA3), thereby induced GATA3-mediated UCP1 upregulation. In addition, to confirm the regulations of UCP1 and GATA3 by ANXA1 *in vivo*, diabetic mice with PTECs-specific deletion of *Anxa1* (Ggt-Cre/*Anxa1*^flox/flox^) were generated, which showed decreased UCP1 and GATA3 expression in their renal cortex with more severe renal injuries. Further investigation revealed that UCP1 deficiency aggravated kidney injuries, including uACR, interstitial inflammation and fibrosis, and enhanced mitochondrial fission in diabetic mice. Mechanistically, UCP1 stabilized cardiolipin contents and reduced mitochondrial fission through the aristaless-related homeobox (ARX)/cardiolipin synthase 1 (CRLS1) pathway. Moreover, specific overexpression of *Crls1* in the renal cortex rescued renal injury and attenuated excessive mitochondrial fission in diabetic *Ucp1*-KO mice. Finally, pharmacological upregulation of UCP1 by CL316243 attenuated established DN in *db*/*db* mice, highlighting the therapeutic potential of targeting UCP1 for DN treatment. Future investigations are needed to explore the broader regulatory landscape of ANXA1 in mitochondrial function and to better understand the cells-specific actions of UCP1 in the kidney. In addition, it will be of interest to evaluate systemic metabolic implications and perform well-designed preclinical and clinical studies to facilitate the translation of UCP1-targeted therapies in DN.

## Materials and methods

### Human renal biopsy samples

Renal biopsy specimens were collected from DN patients, with general data provided in Table 1. More detailed information was provided in the Supplementary Methods. The Declaration of Helsinki was followed, and procedural approval was received from the Ethics Committee of our hospital (No. 2023[135–002]).

**Table 1:** 

**Parameters**	**DN**	**Parameters**	**DN**
Number of patients	30	HbA1c, % (median, IQR)	6.80(6.90,1.0)
Age, years (mean±s.d.)	49.77±12.03	Total cholesterol, mmol/l(mean±s.d.)	4.99±1.67
Sex, M/F	21/9	Triglycerides, mmol/l (median, IQR)	1.93(1.68,1.54)
Diabetes history, years (median, IQR)	12.79(11,8)	HDL-C, mmol/l (mean±s.d.)	1.01±0.29
Type of diabetes, type 1/2	1/29	LDL-C, mmol/l (mean±s.d.)	2.82±1.13
Scr, μmol/l (median, IQR)	187.03(138.05,101.27)	Glomerulosclerosis, % (mean±s.d.)	26.24±19.34
BUN, mmol/l (median, IQR)	12.39(10.62,7.20)	Tubulointerstitial lesions	
eGFR, ml/min/1.73m2 (mean±s.d.)	44.48±22.45	Tubulointerstitial inflammation,	
Urinary protein, g/24h (mean±s.d.)	4.34±2.97	-/+/++/+++	0/14/11/5
Fasting glucose, mmol/l (median, IQR)	7.55(7.53,1.18)	IFTA score, grade 0/1/2/3	0/0/12/18

### Animal studies

Animal experiments were approved by Ethics Committee in Animal Laboratory Center (license number J202139) and were conducted according to National Institutes of Health guidelines. Detail information was provided in the Supplementary Methods.

### Global Anxa1 knockout mice and global Ucp1 knockout mice

Male littermate WT and *Ucp1* KO (*Ucp1*^-/-^) C57BL/6J mice were purchased from GemPharmatech. Male littermate WT and *Anxa1* KO (*Anxa1*^-/-^) C57BL/6J mice were obtained from Cyagen Biosciences. Genotyping was performed by DNA sequencing, using specific primers: *Ucp1*-forward primer: 5’-AAAGCAGTAGGAGACTTCGAGGG-3’; *Ucp1*-reverse primer: 5’-AAGATTCCAGGTAGGTGCTACCAC-3’. WT and KO genotypes were confirmed by the presence or absence of a 286-bp fragment. For transmission electron microscopy (TEM), at least three independent biological replicates per experimental group were analyzed. For biochemical assays, histological analysis, immunohistochemistry, Western blotting, and other experiments, the following group sizes were used: control WT mice (n = 6), control *Ucp1*^−/−^ mice (n = 9), diabetic WT mice (n = 9), and diabetic *Ucp1*^−/−^ mice (n = 6). And for *Anxa1* cohorts, both diabetic WT and *Anxa1*^−/−^ mice included 6 animals per group.

### PTECs-specific Anxa1 knockout mice generation

*Anxa1^fl/fl^* mice were obtained from VIEWSOLID BIOTECH. GGT-Cre (g-glutamyl transferase) mice were obtained from Cyagen Biosciences (Tg (Ggt-cre) M3Egn). More detailed procedures were provided in the Supplementary Methods.

### High-fat diet (HFD)/ streptozotocin (STZ)-induced DN in mice

To induce DN, mice were placed on a HFD and administered STZ *via* intraperitoneal injection. The full protocol was described in the Supplementary Methods.

### Renal cortical adeno-associated virus delivery in mice

Adeno-associated virus serotype 9 (AAV9) for *UCP1*/*CRLS1* overexpression and control virus were obtained from GeneChem Co. (Shanghai, China). More detailed information was provided in the Supplementary Methods. The specific operation method was shown in Fig. 2A.

**Fig. 1:**
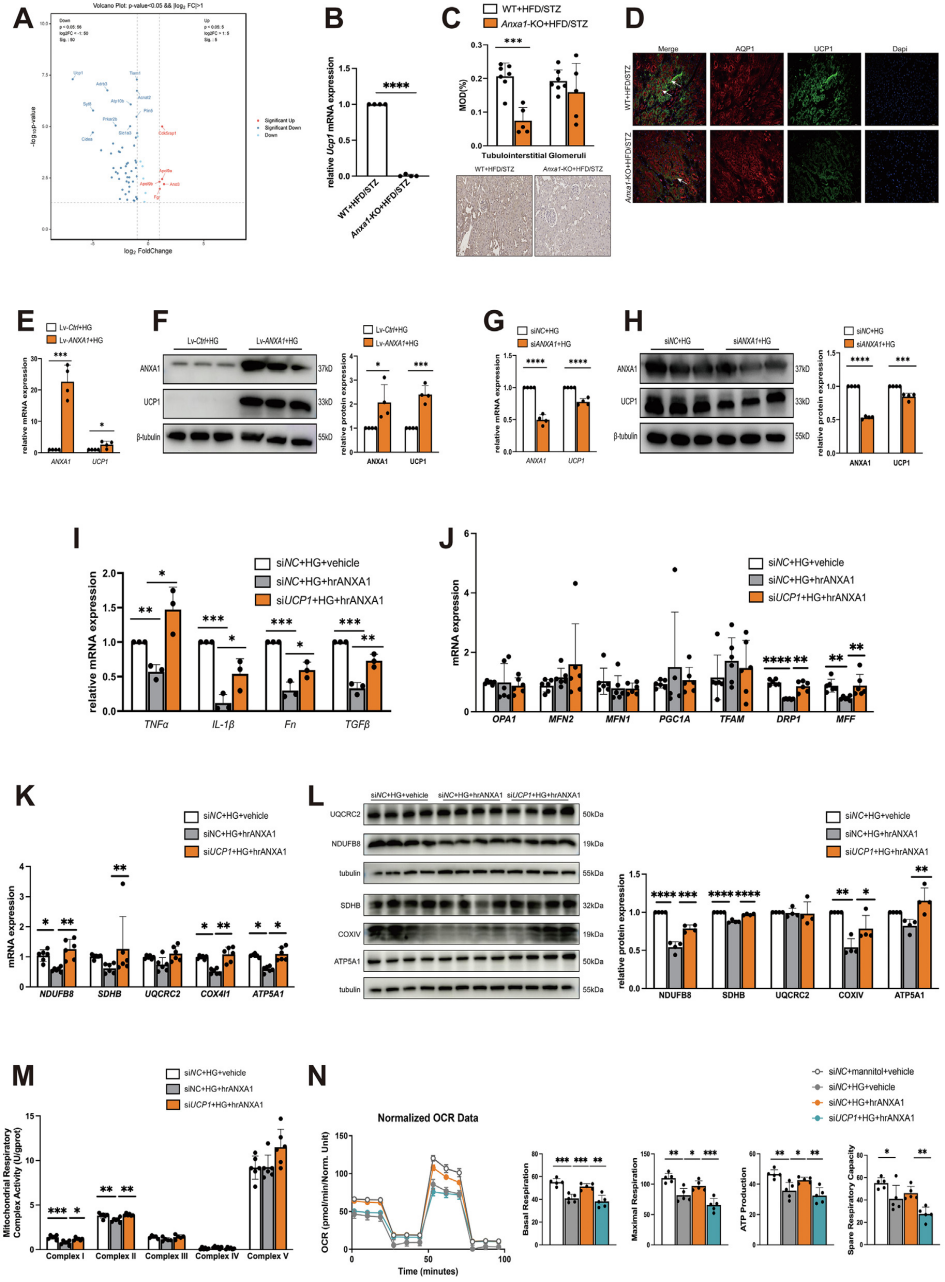
**ANXA1 improved HG-induced mitochondrial dysfunction in HK-2 cells by upregulating UCP1.** (A) Volcano plot showing the expression of transcripts linked to fatty acid oxidation metabolism and mitochondria-associated genes in diabetic *Anxa1^-/-^* mice and diabetic WT mice (n = 3). (B) Relative mRNA level of UCP1 in diabetic *Anxa1^-/-^* mice and diabetic WT mice (n = 4, *p* < 0.0001, two-tailed Student’s *t*-test). (C) Representative IHC images and quantifications of UCP1 in the kidney from diabetic WT mice (n = 7) and diabetic *Anxa1^-/-^* mice (n = 5, *p* = 0.0002, two-tailed Student’s *t*-test). Scale bar:50 μm. (D) Representative IF images of AQP1 (red) and UCP1 (green) in renal tubulointerstitium from diabetic *Anxa1^-/-^* mice and diabetic WT mice. Arrows indicated representative co-localized parts. Scale bar:50 μm. (E) *ANXA1* and *UCP1* mRNA levels in HK-2 cells transfected with lentivirus human *ANXA1* expression plasmid under HG conditions (n = 4 per group, *p* = 0.0002 and *p* = 0.0253, respectively, two-tailed Student’s *t*-test). (F) A representative Western blot (n = 4 blots in total) and quantifications of ANXA1 and UCP1 expression in HK-2 cells transfected with lentivirus human *ANXA1* expression plasmid under HG conditions (*p* = 0.0274 and *p* = 0.0003, respectively, two-tailed Student’s *t*-test). (G) *ANXA1* and *UCP1* mRNA levels in HK-2 cells transfected with *ANXA1* siRNA under HG conditions (n = 4 per group, *p* < 0.0001 and *p* = 0.0001, respectively, two-tailed Student’s *t*-test). (H) A representative Western blot (n = 4 blots in total) and quantifications of ANXA1 and UCP1 expression in HK-2 cells transfected with *ANXA1* siRNA under HG conditions (*p* < 0.0001 and *p* = 0.0009, two-tailed Student’s *t*-test). (I) Relative mRNA level of *TNFα*, *IL-1β*, *Fn* and *TGFβ* (n = 3 per group, si*NC*+HG+vehicle vs. si*NC*+HG+hrANXA1, *p* = 0.0020, *p* = 0.0002, *p* = 0.0006 and *p* = 0.0002, respectively; si*NC*+HG+hrANXA1 vs. si*UCP1*+HG+hrANXA1, *p* = 0.0101, *p* = 0.0428, *p* = 0.0369 and *p* = 0.0054, respectively, one-way ANOVA followed by Tukey’s post hoc test). (J) mRNA level of *OPA1*, *MFN2*, *MFN1*, *PGC1α*, *TFAM*, *DRP1* and *MFF* (n = 6 per group, *DRP1* and *MFF*: si*NC*+HG+vehicle vs. si*NC*+HG+hrANXA1, *p* < 0.0001 and *p* = 0.0023, respectively; si*NC*+ HG+hrANXA1 vs. si*UCP1*+HG+hrANXA1, *p* = 0.0020 and *p* = 0.0021, respectively, one-way ANOVA followed by Tukey’s post hoc test). (K) mRNA level of *NDUFB8*, *SDHB*, *UQCRC2*, *COXAI1* and *ATP5A1* (n = 6 per group, *NDUFB8*, *COXAI1* and *ATP5A*: si*NC*+HG+vehicle vs. si*NC*+HG+hrANXA1, *p* = 0.0421, *p* = 0.0375 and *p* = 0.0434, respectively; si*NC*+HG+hrANXA1 vs. si*UCP1*+HG+hrANXA1, *p* = 0.0017, *p* = 0.0095 and *p* = 0.0224, respectively, one-way ANOVA followed by Tukey’s post hoc test). (L) A representative Western blot (n = 4 blots in total) and quantifications of NDUFB8, SDHB, UQCRC2, COXAI1 and ATP5A1 expression in three groups (NDUFB8, SDHB and COXAI1: si*NC*+HG+vehicle vs. si*NC*+HG+hrANXA1, *p* < 0.0001, *p* < 0.0001, *p* = 0.0011, respectively; si*NC*+HG+hrANXA1 vs. si*UCP1*+HG+hrANXA1, *p* = 0.0002, *p* < 0.0001, *p* = 0.0428, respectively, one-way ANOVA followed by Tukey’s post hoc test). (M) Enzymatic activity assays of mitochondrial Complex I-V in three groups (n = 6 per group, Complex I and II: si*NC*+HG+vehicle vs. si*NC*+HG+hrANXA1, *p* = 0.0005 and *p* = 0.0052, respectively; si*NC*+HG+hrANXA1 vs. si*UCP1*+HG+hrANXA1, *p* = 0.0126 and *p* = 0.0013, respectively, one-way ANOVA followed by Tukey’s post hoc test). (N) OCR in cells exposed to mannitol or HG for 72 h. si*UCP1* and si*NC* cells received hrANXA1 treatment exposed to HG for 72 h (n = 5 per group). *p < 0.05, **p < 0.01, ***p < 0.001, ****p < 0.0001.

**Fig. 2:**
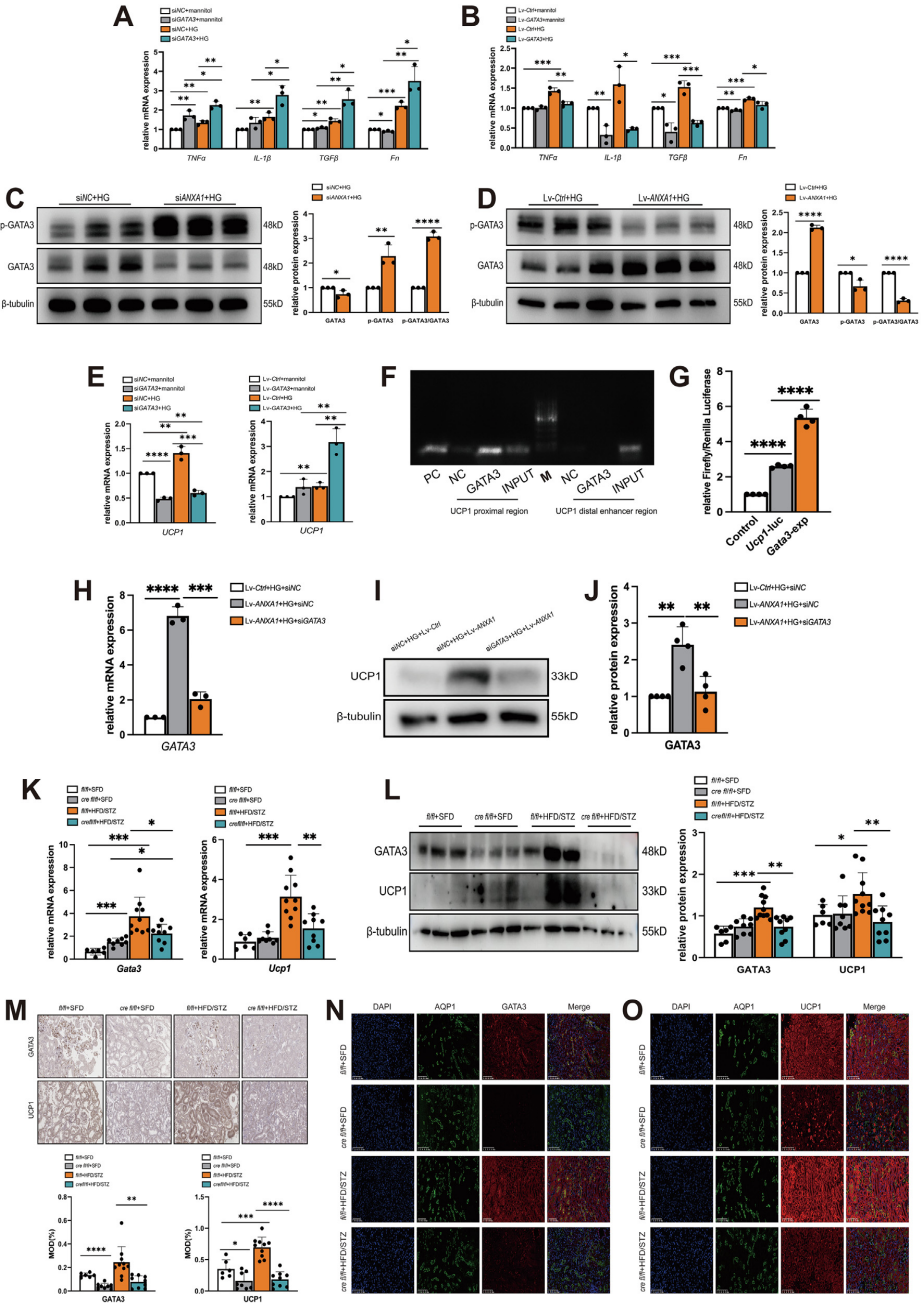
**ANXA1 upregulated UCP1 through stabilizing transcription factor GATA3.** (A) Relative mRNA level of *TNFα*, *IL-1β*, *Fn* and *TGFβ* upon *GATA3* silencing (n = 3 per group, si*NC*+HG vs. si*GATA3*+HG, *p* = 0.0024, *p* = 0.0210, *p* = 0.0439 and *p* = 0.0135, respectively, two-way ANOVA followed by Tukey’s post hoc test). (B) Relative mRNA levels of *TNFα*, *IL-1β*, *Fn* and *TGFβ* upon *GATA3* overexpressing (n = 3 per group, Lv-*Ctrl*+HG vs. Lv-*GATA3*+HG, *p* = 0.0048, *p* = 0.0124, *p* = 0.0488 and *p* = 0.0008, respectively, two-way ANOVA followed by Tukey’s post hoc test). (C) A representative Western blot (n = 3 blots in total) and quantifications of GATA3 and pGATA3 expression in HK-2 cells transfected with si*ANXA1* under HG conditions (si*NC*+HG vs. si*ANXA1*+HG, *p* = 0.0404, *p* = 0.0083 and *p* < 0.0001, respectively, two-tailed Student’s *t*-test). (D) A representative Western blot (n = 3 blots in total) and quantification of GATA3 and pGATA3 expression in HK-2 cells transfected with Lv-*ANXA1* under HG conditions (Lv-*Ctrl*+HG vs. Lv-*ANXA1*+HG, *p* < 0.0001, *p* = 0.0191 and *p* < 0.0001, respectively, two-tailed Student’s *t*-test). (E) Relative mRNA level of *UCP1* upon *GATA3* silencing (n = 3 per group, si*NC*+HG vs. si*GATA3*+HG, *p* = 0.0006, two-way ANOVA followed by Tukey’s post hoc test) and *GATA3* overexpressing (n = 3 per group, Lv-*Ctrl*+HG vs. Lv-*GATA3*+HG, *p* = 0.0053, two-way ANOVA followed by Tukey’s post hoc test). (F) ChIP analysis of murine kidney tubular epithelium (TCMK-1) cells was performed with an anti-GATA3 antibody and the primers for the region of the *UCP1* promoter. PC: positive control (anti-Histone H3 antibody from CST ChIP Kit #9003); NC: negative control (rabbit IgG from CST ChIP Kit #9003); GATA3: anti-GATA3 IP; INPUT: total chromatin. The “M” lane represented DNA marker. (G) Luciferase reporter vectors with *Ucp1* promoter were co-transfected with the vector expressing GATA3 into TCMK-1 cells. Renilla luciferase plasmid was co-transfected as an internal control for normalization. (n = 4 per group, *Ucp1*-luc vs. *Gata3*-exp, *p* < 0.0001, one-way ANOVA followed by Tukey’s post hoc test). The control indicated co-transfection with empty control vectors (con520 as the firefly luciferase vector backbone and con080 as the expression vector backbone). *Ucp1*-luc represented the *Ucp1* promoter-firefly luciferase reporter vector. *Gata3*-exp represented the GATA3 expression vector. (H) Relative mRNA level of *UCP1* after administration of Lv-*ANXA1* in HK-2 cells transfected with si*GATA3* (n = 3 per group, Lv-*ANXA1*+HG+si*NC* vs. Lv-*ANXA1*+HG+si*GATA3*, *p* = 0.0003, one-way ANOVA followed by Tukey’s post hoc test). (I) Representative Western blot images of UCP1 (n = 4, 3 blots in total). (J) Quantifications of Western blot of UCP1 among 3 groups (n = 4 per group, Lv-*ANXA1*+HG+si*NC* vs. Lv-*ANXA1*+HG+si*GATA3*, *p* = 0.0076, one-way ANOVA followed by Tukey’s post hoc test). (K) Relative mRNA levels of GATA3 and UCP1 (n = 6, 8, 10 and 9 respectively, *fl*/*fl*+HFD/STZ vs. *cre fl*/*fl*+HFD/STZ, *p* = 0.0270 and *p* = 0.0025, respectively, two-way ANOVA followed by Tukey’s post hoc test) among 4 groups. (L) A representative Western blot and quantification of GATA3 and UCP1 among 4 groups (n = 6, 8, 10 and 9 in total, respectively, *fl*/*fl*+HFD/STZ vs. *cre fl*/*fl*+HFD/STZ, *p* = 0.0015 and *p* = 0.0012, respectively, two-way ANOVA followed by Tukey’s post hoc test). (M) Representative IHC images and quantification of GATA3 and UCP1 (n = 6, 8, 10 and9 respectively, *fl*/*fl*+HFD/STZ vs. *cre fl*/*fl*+HFD/STZ, *p* = 0.0030, *p* < 0.0001, two-way ANOVA followed by Tukey’s post hoc test) in kidneys of four groups of mice. Scale bar: 50 μm. (N) Representative IF images of AQP1 (green) and GATA3 (red) in the kidneys of 4 mice groups (n = 6, 8, 10, 9 respectively). Scale bar: 100 μm. (O) Representative IF images of AQP1 (green) and UCP1 (red) in the kidneys of 4 groups of mice (n = 6, 8, 10 and 9 respectively). Scale bar: 100 μm. *p < 0.05, **p < 0.01, ***p < 0.001, ****p < 0.0001.

### Administration of CL316243 in db/db mice

At 8 weeks of age, *db/db* mice were administrated CL316243 (3mg/kg)/vehicle (methylcellulose) for 4 weeks (every 9:00 a.m., gavage). Age-matched *db/m* mice were used as controls. CL316243 (MedChemExpress) was provided with a certificate of analysis indicating a purity of 98.57%.

### Cell culture and treatment

The murine kidney tubular epithelial cell line (TCMK-1), immortalized human proximal tubular epithelial cells (HK-2 cells) and primary murine PTECs (MPTECs) were obtained from Fuheng Biotechnology Inc. (China), the American Type Culture Collection (ATCC), and Procell Inc.(China), respectively. To mimic the diabetic microenvironment, cells were cultured in medium containing HG at a final concentration of 30 mmol/L for 72 hours. Detailed treatment protocols were provided in the Supplementary Methods.

### Histological analysis of renal samples

For renal histology assessment, paraffin-embedded kidney sections were stained using conventional histological techniques. Detailed procedures and statistical analysis were provided in the Supplementary Methods.

### Immunofluorescence (IF) and immunohistochemistry (IHC)

IF and IHC were performed as described in the Supplementary Methods.

### TEM

Mitochondrial morphology was analyzed using TEM, with aspect ratio, factor form, circularity and roundness calculated by ImageJ software according to published methods [20]. At least 120 mitochondria were determined in each condition. Detailed protocols were provided in the Supplementary Methods.

### Real-time quantitative polymerase chain reaction (qPCR) analysis

Real-time qPCR was conducted according to standard protocols, with primer sequences shown in Table 2. Detailed information was provided in the Supplementary Methods.

**Table 2:** 

Gene	Primer Sequence 5' to 3'	
	Forward	Reverse
Human		
UCP1	CAGGGAAAGAAACAGCACCT	GGCTCTGTGCTTGAAGTCTG
TNFA	CCTCTCTCTAATCAGCCCTCTG	GAGGACCTGGGAGTAGATGAG
Fn	GGTACAGGGTGACCTACTCG	GGAATAGCTGTGGACTGGGT
TGFB	CAGCAACAATTCCTGGCGAT	TGAACCCGTTGATGTCCACT
IL1B	GCTGAGGAAGATGCTGGTTC	TCCATATCCTGTCCCTGGAG
ANXA1	CTCCAGGAAACAGGAAAGCC	TCAGTTCCAAGGCCCTTCAT
MFF	GAGCAGTTGGCAGACTAAAAAGA	GGTGCAGAAATCGGTGCCT
DRP1	TTTGACACTTGTGGATTTGCCA	AGTGACAGCGAGGATAATGGA
OPA1	TGTGAGGTCTGCCAGTCTTTA	TGTCCTTAATTGGGGTCGTTG
MFN1	TGGCTAAGAAGGCGATTACTGC	TCTCCGAGATAGCACCTCACC
MFN2	GGCCCAACTCTAAGTGCCC	AAGTGCTTTTCCGTCTGCATC
PGC1α	GCTTTCTGGGTGGACTCAAGT	GAGGGCAATCCGTCTTCATCC
TFAM	ATGGCGTTTCTCCGAAGCAT	TCCGCCCTATAAGCATCTTGA
PINK1	GCCTCATCGAGGAAAAACAGG	GTCTCGTGTCCAACGGGTC
PRKN	CCCACCTCTGACAAGGAAACA	TCGTGAACAAACTGCCGATCA
CRLS1	AGAGATGTAATGTTGATTGCTGCTG	TGCCACCAAGATTAACTGGACTG
GATA3	GCCCCTCATTAAGCCCAAG	TTGTGGTGGTCTGACAGTTCG
actinb	GAAGTGTGACGTGGACATCC	CCGATCCACACGGAGTACTT
18s	GTAACCCGTTGAACCCCATTC	GCCTCACTAAACCATCCAATCG
Mouse		
Ucp1	AGCTTTGCCTCACTCAGGAT	AGAGGCAGGTGTTTCTCTCC
Ucp2	GCCTCTGGAAAGGGACTTCT	CGAAGGCAGAAGTGAAGTGG
Ucp3	CAAAGGATTTGTGCCCTCCT	GCTTTGTTCTGTTCCAGGCA
Adrp	AAGGTGAAGAGCATCATAACCCT	TCACGCCTTTCATAACACATTCC
Col1a1	ATCTCCTGGTGCTGATGGAC	ACCTTGTTTGCCAGGTTCAC
Mff	AGCTGCCGCCACTTCTAATC	TGCATCTACCACAGTCATGTCA
Drp1	CCTCAGATCGTCGTAGTGGGA	GTTCCTCTGGGAAGAAGGTCC
Opa1	CGACTTTGCCGAGGATAGCTT	CGTTGTGAACACACTGCTCTTG
Mfn1	ATGGCAGAAACGGTATCTCCA	CTCGGATGCTATTCGATCAAGTT
Mfn2	CTGGGGACCGGATCTTCTTC	CTGCCTCTCGAAATTCTGAAACT
Cd68	TGTCTGATCTTGCTAGGACCG	GAGAGTAACGGCCTTTTTGTGA
Kim1	GGAATCCCATCCCATACTCCT	AAGTATGTACCTGGTGATAGCCAC
Asma	GAGGCACCACTGAACCCTAA	CATCTCCAGAGTCCAGCACA
F4/80	TGACTCACCTTGTGGTCCTAA	CTTCCCAGAATCCAGTCTTTCC
Gata3	CTCGGCCATTCGTACATGGAA	GGATACCTCTGCACCGTAGC
Ngal	CCATCTATGAGCTACAAGAGAACAAT	TCTGATCCAGTAGCGACAGC
Crls1	ATCAGCTTTGGGAAGTGCTC	ACCTTGCTGATGAATGTTGGT
Arx	GGCCGGAGTGCAAGAGTAAAT	TGCATGGCTTTTTCCTGGTCA
Tfap2c	ATCCCTCACCTCTCCTCTCC	CCAGATGCGAGTAATGGTCGG
Zfp161	AGTAGACAGCTCGTCCGTCAT	CGGATACCGAGAATCTGCCC
18s	GTAACCCGTTGAACCCCATTC	GCCTCACTAAACCATCCAATCG

### Measurement of intracellular ROS, lipid peroxidation and cardiolipin

Intracellular ROS levels, lipid peroxidation and cardiolipin content were assessed using specific fluorescent probes and analyzed by high-resolution microscopy and flow cytometry, as detailed in the Supplementary Methods.

### Western blot

Western blot was conducted according to standard protocols. Detailed protocols were provided in the Supplementary Methods.

### Isolation of primary MPETCs

Primary MPTECs were isolated from 3-week-old mice according to published methods [21] with some modifications. Detailed surgical procedures were provided in the Supplementary Methods.

### Biochemical analysis of serum and urine samples

Biochemical analysis of serum and urine samples was performed using standard protocols. Detailed information was provided in the Supplementary Methods.

### Mitochondrial visualization

Mitochondrial morphology was visualized by MitoTracker (Beyotime) based on published methods [22,23]. Detailed information was provided in the Supplementary Methods.

### Mitochondrial complexes enzymatic activity assays

Activities of mitochondrial respiratory chain Complex (I-V) were measured using colorimetric assay kits (Elabscience) according to the manufacturer’s instructions. Briefly, mitochondria were isolated from HK-2 cells using a mitochondrial isolation kit and quantified for protein concentration using a BCA assay. The enzymatic activity of Complex I was assessed by NADH oxidation (340 nm), Complex II by DCPIP reduction (600 nm), Complex III by cytochrome c reduction (550 nm), Complex IV by cytochrome c oxidation (550 nm), and Complex V by ATP hydrolysis. Absorbance changes were recorded with a microplate reader and normalized to mitochondrial protein content.

### Oxygen consumption rate (OCR) assay

OCR assay was conducted by standard protocols. More details were provided in the Supplementary Methods.

### Oil red O staining

Oil red O staining was conducted following standard protocols. Detailed protocols were provided in the Supplementary Methods.

### Co-immunoprecipitation (Co-IP)

Co-IP was conducted by according to the manufacturer’s instructions. Details procedures were provided in the Supplementary Methods.

### Chromatin immunoprecipitation (ChIP) assay

ChIP assay was performed according to instructions (Cell Signaling Technology, CST) with modifications as described in published methods [24]. Details procedures were provided in the Supplementary Methods.

### Luciferase assay

Detailed protocols of luciferase assay were provided in the Supplementary Methods.

### Lentivirus-mediated gene expression and interference

Details about lentivirus-mediated gene expression and interference were provided in the Supplementary Methods.

### Small interfering RNA (siRNA)-mediated gene knockdown and transfections

Details regarding siRNA-mediated gene knockdown and transfections were provided in the Supplementary Methods.

### Cardiolipin content analysis

Cardiolipin levels were quantified using Bligh and Dyer’s lipid extraction method, with some modifications as previously described [25]. Detailed information was provided in the Supplementary Methods.

### Non-targeted metabolomics

Kidney cortex tissue was homogenized and processed for non-targeted metabolomics analysis, with detailed information available in the Supplementary Methods.

### Statistical analysis

Data analysis was performed using SPSS statistical software (v24.0, NY) or GraphPad Software (v8.0, San Diego, CA). Continuous data were expressed as mean ± SD or median and interquartile range (IQR) as appropriate. Differences between the two groups were analyzed using a two-tailed Student’s *t-test* or Mann-Whitney U test, depending on data normality and homogeneity of variance. For comparisons among multiple groups, one-way or two-way ANOVA with Tukey’s post hoc test was used, depending on the number of independent variables. Statistical significance was considered for *p*-value < 0.05. All details about statistics regarding *p*-value and *n* (numbers) were provided in main and Supplementary Figure Legends.

## Results

### ANXA1 improved HG-induced mitochondrial dysfunction in PTECs by upregulating UCP1

In our previous study [11], *Anxa1*-KO mice were used in the DN model induced by HFD/STZ to investigate the protective role of ANXA1. In the current study, volcano plot analysis revealed that genes involved in FAO metabolism and mitochondria-associated were the most significantly downregulated in diabetic *Anxa1^-/-^* mice compared with diabetic WT mice. Among these genes, *Ucp1* was the most significantly differentially expressed one (Fig. 1A). Consistently, *Ucp1* was decreased in the renal cortex of diabetic *Anxa1^-/-^* mice at the mRNA level (Fig. 1B, two-tailed Student’s *t*-test). Moreover, immunohistochemistry confirmed that the expression of UCP1 was downregulated in diabetic *Anxa1^-/-^* mice (n = 5) compared with diabetic WT mice (n = 7), especially in the tubulointerstitium (Fig. 1C, *p*  = 0.0002, two-tailed Student’s *t*-test). More specifically, the expression of UCP1 was significantly reduced in PTECs (marked as aquaporin 1 [AQP1]) of diabetic *Anxa1^-/-^* mice (Fig. 1D).

To clarify the temporal dynamics of the ANXA1-UCP1 axis, a time-course experiment under HG conditions (30 mM) for 48 and 72 hours was conducted in both HK-2 cells and primary MPTECs. The results showed that both *ANXA1/Anxa1* and *UCP1/Ucp1* levels progressively increased over time, reaching peak at 72 hours. In parallel, HG treatment also led to time-dependent elevations in pro-inflammatory factors including tumor necrosis factor alpha (*TNFα/Tnfα*), interleukin-1beta (*IL-1β*), and EGF-like module-containing mucin-like hormone receptor-like 1 (*F4/80*), fibrotic markers including fibronectin (*FN*), transforming growth factor beta (*TGFβ*) and α-smooth muscle actin (*αSMA*), tubular injury markers including kidney injury molecule-1 (*KIM-1/Kim-1*) and neutrophil gelatinase-associated lipocalin (*NGAL/Ngal*), indicating cumulative cellular stress and injury following prolonged exposure to hyperglycemia (Supplementary Fig. S1A, S1B).

*In vitro*, the lentiviral system was used to overexpress *ANXA1* in HK-2 cells. Under HG conditions, *UCP1* expression was significantly upregulated in ANXA1-overexpressing cells compared to control cells, at both the mRNA and protein levels. (Fig. 1E, *p* = 0.0002, *p* = 0.0253, respectively, two-tailed Student’s *t*-test; Fig. 1F, *p* = 0.0274, *p* = 0.0003, respectively, two-tailed Student’s *t*-test). Under HG conditions, the expression of UCP1 was significantly decreased in si*ANXA1*-treated HK-2 cells at both the mRNA and protein levels, compared with si*NC*-treated HK-2 cells (Fig. 1G, *p* < 0.0001, *p* = 0.0001, respectively, two-tailed Student’s *t*-test; Fig. 1H, *p* < 0.0001, *p* = 0.0009, two-tailed Student’s *t*-test). Consistent with the findings in HK-2 cells, under HG conditions, the expression of UCP1 was also significantly decreased in si*Anxa1*-treated primary MPTECs at both the mRNA and protein levels, compared with si*Nc* controls (Fig. S1C, S1D). HG-induced expression of proinflammatory factors including *TNFα* and *IL-1β*, and profibrotic factors including *Fn* and *TGFβ* in HK-2 cells, was downregulated by hrANXA1, and these effects were abolished upon *UCP1* silencing (Fig. 1I). Besides, HG-induced expression of *Tnfα*, *F4/80, αSMA*, *Kim-1* and *Ngal* was decreased by hrANXA1, and such protective effects were abolished upon *Ucp1* silencing in primary MPTECs (Fig. S1E). In HG-treated HK-2 cells, compared with vehicle treatment, hrANXA1 treatment significantly downregulated mRNA levels of mitochondrial fission-related genes, including mitochondrial fission factor (*MFF*, *p* = 0.0023, one-way ANOVA followed by Tukey’s post hoc test) and dynamin-related protein 1 (*DRP1*, *p* < 0.0001, one-way ANOVA followed by Tukey’s post hoc test). However, these effects were abolished upon *UCP1* silencing (*MFF*, *p* = 0.0021; *DRP1*, *p* = 0.0020, n = 6 per group, respectively, one-way ANOVA followed by Tukey’s post hoc test). In HG-treated HK-2 cells, hrANXA1 treatment did not have an obvious effect on mRNA levels of mitochondrial fusion-related genes, including mitofusin 1 (*MFN1*), mitofusin 2 (*MFN2*), and optic atrophy 1 (*OPA1*), or mitochondrial biogenesis-related genes, including peroxisome proliferator-activated receptor gamma coactivator 1-alpha (*PGC1α*) and mitochondrial transcription factor A (*TFAM*), compared with vehicle-treated control group (Fig. 1J). Furthermore, in HG-treated HK-2 cells, compared with vehicle treatment, hrANXA1 treatment significantly decreased mRNA levels of mitochondrial respiratory chain Complexes I, IV, and V (represented by their core subunits *NDUFB8*, *p* = 0.0421; *COXIV*, *p* = 0.0375; and *ATP5A1*, *p* = 0.0434, respectively, one-way ANOVA followed by Tukey’s post hoc test), and significantly decreased protein levels of Complexes I, II and IV (represented by their core subunits NDUFB8, *p* < 0.0001; SDHB, *p* < 0.0001; COXIV, *p* = 0.001, respectively, one-way ANOVA followed by Tukey’s post hoc test); however, these effects were significantly abolished upon *UCP1* silencing (*NDUFB8*, *p* = 0.0017; *COXIV*, *p* = 0.0095; and *ATP5A1*, *p* = 0.0224, in mRNA levels; and NDUFB8, *p* = 0.0002; SDHB, *p* < 0.0001; COXIV, *p* = 0.0428, in protein levels, respectively, one-way ANOVA followed by Tukey’s post hoc test) (Fig. 1K, 1L). The enzymatic activities of Complexes I-V in HK-2 cells under HG conditions were further investigated. Compared with vehicle treatment, hrANXA1 treatment significantly reduced the activities of Complex I (NADH dehydrogenase, *p* = 0.0005, one-way ANOVA followed by Tukey’s post hoc test) and Complex II (succinate dehydrogenase, *p* = 0.0052, one-way ANOVA followed by Tukey’s post hoc test), while the activities of Complex III-V did not change obviously. Notably, the effects of hrANXA1 on enzymatic activities of Complex I and II were significantly abolished (NADH dehydrogenase, *p* = 0.0126; succinate dehydrogenase, *p* = 0.0013, one-way ANOVA followed by Tukey’s post hoc test) upon *UCP1* silencing (Fig. 1M). OCR was measured in HK-2 cells to assess mitochondrial respiratory function across experimental groups. It was found that HG treatment suppressed the basal respiration, maximal respiration, respiratory capacity and ATP production, all of which were restored by hrANXA1 treatment. However, these protective effects of hrANXA1 were abolished upon *UCP1* silencing (Fig. 1N). Besides, these effects on OCR were consistently observed in primary MPTECs (Fig. S1F).

Collectively, these results demonstrated that ANXA1 could ameliorate HG-induced mitochondrial dysfunction by upregulating UCP1 in PTECs.

### ANXA1 upregulated UCP1 through stabilizing transcription factor GATA3

To determine whether ANXA1 directly interacts with UCP1, reciprocal Co-IP experiments were performed. However, no evidence of such an interaction was observed (Fig. S2A). It was reported that ANXA1 has a mutually regulatory relationship with the transcription factor GATA3 [26,27]. In kidneys of diabetic *Anxa1^-/-^* mice, *GATA3* was significantly downregulated compared with diabetic WT mice in transcriptomics analysis (Fig. S2B). Consistently, under HG conditions, the mRNA level of *GATA3* was decreased in si*ANXA1*-treated HK-2 cells and was increased in Lv-*ANXA1*-treated HK-2 cells (Fig. S2C). In immunohistochemical staining, the level of GATA3 was higher in kidneys of DN patients than in healthy controls (Fig. S2D). *In vitro*, HG-induced upregulation of *TNFα*, *IL-1β*, *TGFβ* and *Fn* in HK-2 cells was exacerbated upon *GATA3* knockdown and was mitigated upon *GATA3* overexpression (Fig. 2A, *p* = 0.0024, *p* = 0.0210, *p* = 0.0439, *p* = 0.0135, respectively; 2B, *p* = 0.0048, *p* = 0.0124, *p* = 0.0488, *p* = 0.0008, respectively, two-way ANOVA followed by Tukey’s post hoc test). It was reported that GATA3 could be phosphorylated at Ser308 (pGATA3) and then be degraded by proteasomes [28,29]. It was further confirmed that pGATA3 was cleaved by proteasomes in HK-2 cells (Fig. S2E). In HK-2 cells treated by HG, si*ANXA1* group had decreased levels of total GATA3 and increased the pGATA3/GATA3 ratio compared with si*NC* group (Fig. 2C, *p* = 0.0404, *p* = 0.0083, *p* < 0.0001, respectively, two-tailed Student’s *t*-test). Consistently, total GATA3 was significantly increased, while the pGATA3/GATA3 ratio was significantly decreased upon overexpression of *ANXA1*(Fig. 2D, *p* < 0.0001, *p* = 0.0191, *p* < 0.0001, respectively, two-tailed Student’s *t*-test). Additionally, immunoprecipitation (IP) assays demonstrated that the ubiquitination levels of GATA3 were significantly increased upon *ANXA1*/*Anxa1* silencing in both cell types under HG conditions (Fig. S2F). By mass spectrometry and IP, ANXA1 was found to bind to thioredoxin (TRX) (Fig. S2G, Fig. S2H). Under HG conditions, GATA3 expression was significantly higher in Lv-*ANXA1*-treated HK-2 cells than in Lv-*Ctrl* controls. However, this increase was abolished upon thioredoxin knockdown, suggesting that ANXA1 may stabilize GATA3 through binding to thioredoxin (Fig. S2I). The apoptosis signal-regulating kinase 1 (ASK1)/mitogen-activated protein kinase (MAPK) pathway has been previously implicated as a potential mechanism underlying thioredoxin-mediated stabilization of GATA3 [26]. To further substantiate this mechanism, whether pharmacological inhibition of the ASK1/MAPK pathway could restore GATA3 stability under thioredoxin deficiency were investigated. In si*TRX*-transfected HK-2 cells exposed to HG, the ratio of p-GATA3 to total GATA3 expression was decreased by combined treatment with the ASK1 inhibitor GS444217 and the MAPK inhibitor SB203580, compared with the vehicle control, indicating a synergistic rescue effect on GATA3 stability (Fig. S2J). *UCP1* mRNA expression was significantly decreased following *GATA3* knockdown and, conversely, significantly increased upon *GATA3* overexpression under both HG and mannitol (osmotic) control conditions in HK-2 cells (Fig. 2E, *p* = 0.0006, *p* = 0.0053, respectively, two-way ANOVA followed by Tukey’s post hoc test). *Ucp1* contains two regulatory regions, including a proximal promoter and a distal enhancer [30,31]. ChIP assays showed GATA3 directly bound to the proximal promoter region of *Ucp1*, but not to the distal enhancer region (Fig. 2F). Consistently, dual-luciferase reporter assays showed that ectopic expression of GATA3 significantly increased *Ucp1* promoter activity. Firefly luciferase signals were normalized to Renilla internal control and compared with the corresponding empty-vector groups, suggesting that GATA3 enhanced the transcriptional activity of *Ucp1* (Fig. 2G, *p* < 0.0001, one-way ANOVA followed by Tukey’s post hoc test). Finally, overexpression of *ANXA1* upregulated UCP1 at both the mRNA (Fig. 2H, *p* = 0.0003, one-way ANOVA followed by Tukey’s post hoc test) and protein levels (Fig. 2I, 2J, *p* = 0.0076, one-way ANOVA followed by Tukey’s post hoc test) under HG conditions, but these effects were abolished by knockdown of *GATA3*.

PTECs-specific *Anxa1* knockout (*Ggt*-*Cre*/*Anxa1 ^fl/fl^*) mice following a *Cre*-*LoxP* recombination system with *Ggt*-*Cre* were generated. The genotype identification and validation of *Anxa1* knockout efficiency were detected (Fig. S2K). ANXA1 protein was almost absent in the knockout lanes, and residual background signals were regarded as “undetectable”, indicating efficient knockout and near-complete loss of protein expression. DN was established in *Ggt*-*Cre*/*Anxa1 ^fl/fl^* mice and *Anxa1^fl/fl^* mice as described above. Compared with diabetic *Anxa1^fl/fl^* mice, more severe renal injuries were exhibited in diabetic *Ggt*-*Cre*/*Anxa1 ^fl/fl^* mice, including an elevated uACR (330.0 ± 295.0 vs. 71.94 ± 54.62 μg/mg, *p* = 0.0128), increased interstitial fibrosis and tubular atrophy (IFTA) score (Fig. S2L). Besides, the expression of both GATA3 and UCP1 was significantly decreased in diabetic *Ggt*-*Cre*/*Anxa1 ^fl/fl^* mice at both the mRNA and protein levels compared with diabetic *Anxa1^fl/fl^* mice (Fig. 2K, *p* = 0.0270, *p* = 0.0025, respectively, two-way ANOVA followed by Tukey’s post hoc test; 2L, *p* = 0.0015, *p* = 0.0012, respectively, two-way ANOVA followed by Tukey’s post hoc test). Consistently, immunohistochemical staining of renal tissues revealed markedly reduced expression of both GATA3 and UCP1 in diabetic *Ggt*-*Cre*/*Anxa1 ^fl/fl^* mice compared with diabetic *Anxa1^fl/fl^* mice (Fig. 2M, *p* = 0.0030, *p* < 0.0001, respectively, two-way ANOVA followed by Tukey’s post hoc test). More specifically, compared with diabetic *Anxa1^fl/fl^* mice, the expression of both GATA3 and UCP1 was significantly decreased (Fig. S2M) in PTECs (marked as AQP1) of diabetic *Ggt*-*Cre*/*Anxa1 ^fl/fl^* mice, as revealed by fluorescent staining experiments (Fig. 2N, 2O).

Collectively, these results demonstrated that ANXA1 regulated UCP1 expression through stabilizing GATA3.

### Renal overexpression of *Ucp1* rescued kidney injuries in HFD/STZ-induced diabetic *Anxa1^-/-^* mice

**Fig. 3:**
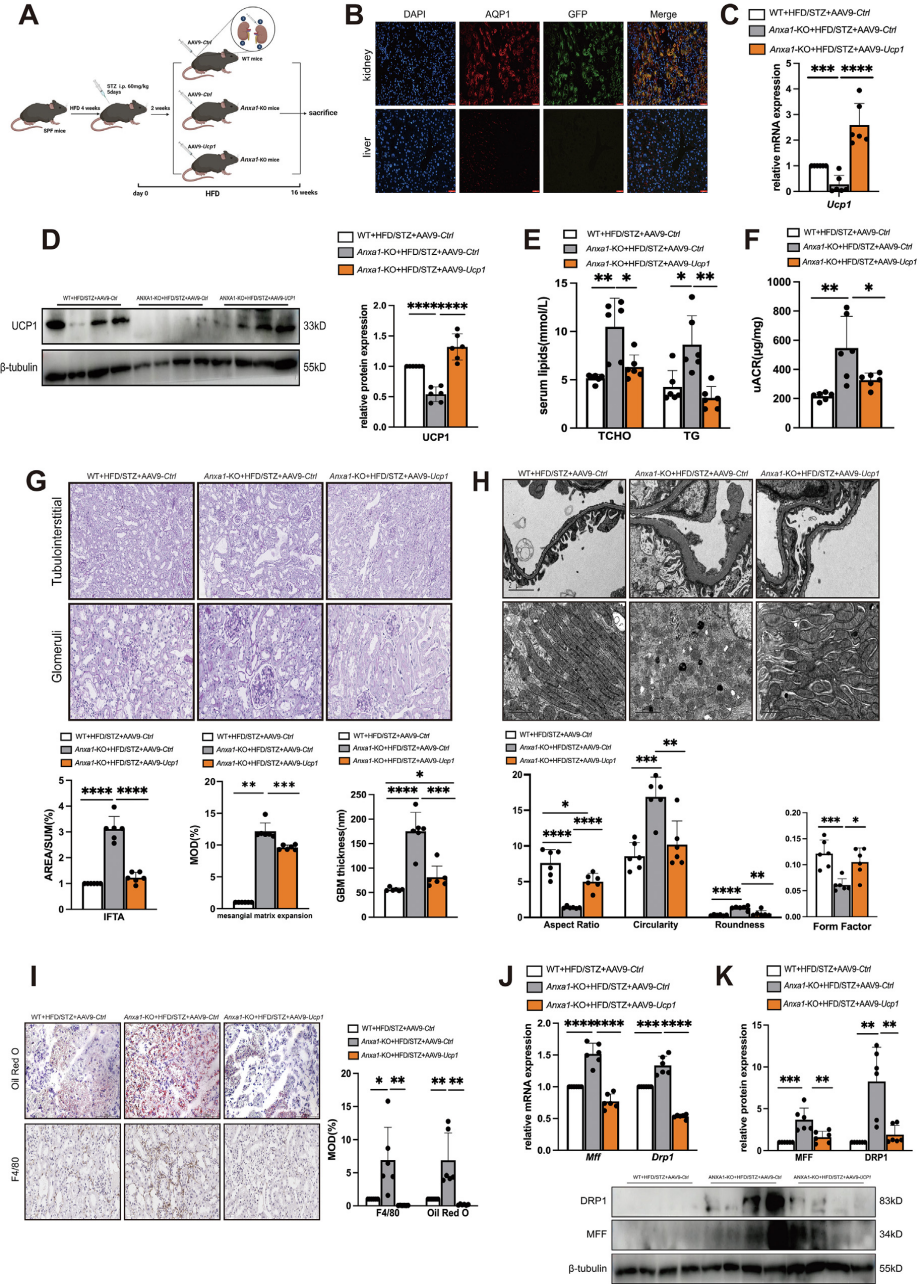
**Renal overexpression of *Ucp1* rescued kidney injuries in HFD/STZ-induced diabetic *Anxa1^-/-^* mice.** (A) A schematic diagram showing the procedure of renal cortical adeno-associated virus serotype 9 (AAV9) delivery in HFD/STZ-induced diabetic *Anxa1^-/-^* mice. (B) Fluorescent photomicrographs of the kidney and liver were taken after AAV9 delivery in mice renal cortex. Scale bar:50 μm. (C) Determination of *Ucp1* overexpression efficiency by qPCR (n = 6 per group, *Anxa1*-KO+HFD/STZ+AAV9-*Ctrl* vs. *Anxa1*-KO+HFD/STZ+AAV9-*Ucp1*, *p* = 0.0015, one-way ANOVA followed by Tukey’s post hoc test). (D) Determination of *Ucp1* overexpression efficiency by Western blot (n = 6 per group, *Anxa1*-KO+HFD/STZ+AAV9-*Ctrl* vs. *Anxa1*-KO+HFD/STZ+AAV9-*Ucp1*, *p* < 0.0001, one-way ANOVA followed by Tukey’s post hoc test). (E) Serum total triglyceride (TG) and cholesterol (TCHO) concentrations in mice (n = 6 per group, *Anxa1*-KO+HFD/STZ+AAV9-*Ctrl* vs. *Anxa1*-KO+HFD/STZ+AAV9-*Ucp1*, *p* = 0.0185, *p* = 0.0002, respectively, one-way ANOVA followed by Tukey’s post hoc test). (F) Urinary albumin-to-creatinine ratio (uACR) in mice (n = 6 per group, *Anxa1*-KO+HFD/STZ+AAV9-*Ctrl* vs. *Anxa1*-KO+HFD/STZ+AAV9-*Ucp1*, *p* = 0.0267, one-way ANOVA followed by Tukey’s post hoc test). (G) Representative images and quantitative analysis of IFTA score and degree of mesangial matrix expansion of morphological examination by Periodic acid-Schiff (PAS) (n = 6 mice per group, *Anxa1*-KO+HFD/STZ+AAV9-*Ctrl* vs. *Anxa1*-KO+HFD/STZ+AAV9-*Ucp1*, *p* < 0.0001, *p* = 0.0001, respectively, one-way ANOVA followed by Tukey’s post hoc test). Scale bar:50 μm. (H) Quantitative analysis of TEM analysis showed GBM thickness and mitochondrial morphology (n = 6 per group, *Anxa1*-KO+HFD/STZ+AAV9-*Ctrl* vs. *Anxa1*-KO+HFD/STZ+AAV9-*Ucp1*, *p* = 0.0005, *p* < 0.0001, *p* = 0.0042, *p* = 0.0036 and *p* = 0.0025, respectively, one-way ANOVA followed by Tukey’s post hoc test) in mice. Scale bar: 2 μm. (I) Representative images and quantitative analysis of Oil Red O and F4/80 staining in mice renal cortex (n = 6 per group, *Anxa1*-KO+HFD/STZ+AAV9-*Ctrl* vs. *Anxa1*-KO+HFD/STZ+AAV9-*Ucp1*, *p* = 0.0070, *p* = 0.0026, one-way ANOVA followed by Tukey’s post hoc test). Scale bar:100 μm, 50μm respectively. (J) Relative mRNA level of *MFF*, *DRP1* (n = 6 per group, *Anxa1*-KO+HFD/STZ+AAV9-*Ctrl* vs. *Anxa1*-KO+HFD/STZ+AAV9-*Ucp1*, *p* < 0.0001 and *p* < 0.0001, respectively, one-way ANOVA followed by Tukey’s post hoc test). (K) A representative Western blot (n = 6 blots in total) and quantification of MFF and DRP1 among 3 mice groups (*Anxa1*-KO+HFD/STZ+AAV9-*Ctrl* vs. *Anxa1*-KO+HFD/STZ+AAV9-*Ucp1*, *p* = 0.0034 and *p* = 0.0073, respectively, one-way ANOVA followed by Tukey’s post hoc test). *p < 0.05, **p < 0.01, ***p < 0.001, ****p < 0.0001.

Since ANXA1 exerted its protective role through UCP1 in PTECs, whether *Ucp1* overexpression could rescue the renal phenotype in diabetic *Anxa1^-/-^* mice was investigated. Based on the abovementioned HFD/STZ-induced diabetic *Anxa1^-/-^* mice model, the AAV9 overexpressing *Ucp1* (AAV9-*Ucp1*) or the AAV9-*Ctrl* was injected directly into the renal cortex fifteen days after STZ injections (Fig. 3A). The fluorescence intensity in the renal cortex of injected mice was significantly higher than that in the liver, indicating kidney-targeted delivery of AAV9 (Fig. 3B). It was found that UCP1 was upregulated significantly at both the mRNA and protein levels in the AAV9-*Ucp1* group compared to the AAV9-*Ctrl* group (Fig. 3C, *p* = 0.0015, one-way ANOVA followed by Tukey’s post hoc test; 3D, *p* < 0.0001, one-way ANOVA followed by Tukey’s post hoc test). Compared with the AAV9-*Ctrl* group, renal overexpression of *Ucp1* significantly decreased serum triglyceride (TG) and total cholesterol (TCHO) (Fig. 3E, *p* = 0.0185 and *p* = 0.0002, respectively, one-way ANOVA followed by Tukey’s post hoc test), and obviously rescued the renal phenotype of diabetic *Anxa1^-/-^* mice, as manifested as reduction of uACR (327.0 ± 48.66 vs. 546.6 ± 218.3 μg/mg, *p* = 0.0267, one-way ANOVA followed by Tukey’s post hoc test, Fig. 3F), IFTA score, and the degree of mesangial matrix expansion (Fig. 3G, *p* < 0.0001, *p* = 0.0001, respectively, one-way ANOVA followed by Tukey’s post hoc test). TEM suggested that compared with the AAV9-*Ctrl* group, renal overexpression of *Ucp1* significantly reduced GBM thickening and alleviated mitochondrial swelling and disorganization induced by *Anxa1* knockout (Fig. 3H, *p* = 0.0005, *p* < 0.0001, *p* = 0.0042, *p* = 0.0036 and *p* = 0.0025, respectively, one-way ANOVA followed by Tukey’s post hoc test). Furthermore, the reduction of F4/80 and Oil red O staining suggested that renal overexpression of *Ucp1* also mitigated renal inflammation and lipid deposition induced by *Anxa1* knockout (Fig. 3I, *p* = 0.0070 and *p* = 0.0026, respectively, one-way ANOVA followed by Tukey’s post hoc test). *Anxa1* deficiency aggravated mitochondrial fission in diabetic mice in the renal cortex, as shown by increased mRNA and protein levels of MFF and DRP1, while overexpression of *Ucp1* significantly attenuated such increasing (Fig. 3J, *p* < 0.0001 and *p* < 0.0001, respectively, one-way ANOVA followed by Tukey’s post hoc test; 3K, *p* = 0.0034 and *p* = 0.0073, respectively, one-way ANOVA followed by Tukey’s post hoc test).

Collectively, renal overexpression of *Ucp1* ameliorated kidney injury and restored mitochondrial homeostasis in diabetic *Anxa1*^−/−^ mice, rescuing the detrimental effects of ANXA1 deficiency.

### UCP1 deficiency aggravated renal injury in diabetes

**Fig. 4:**
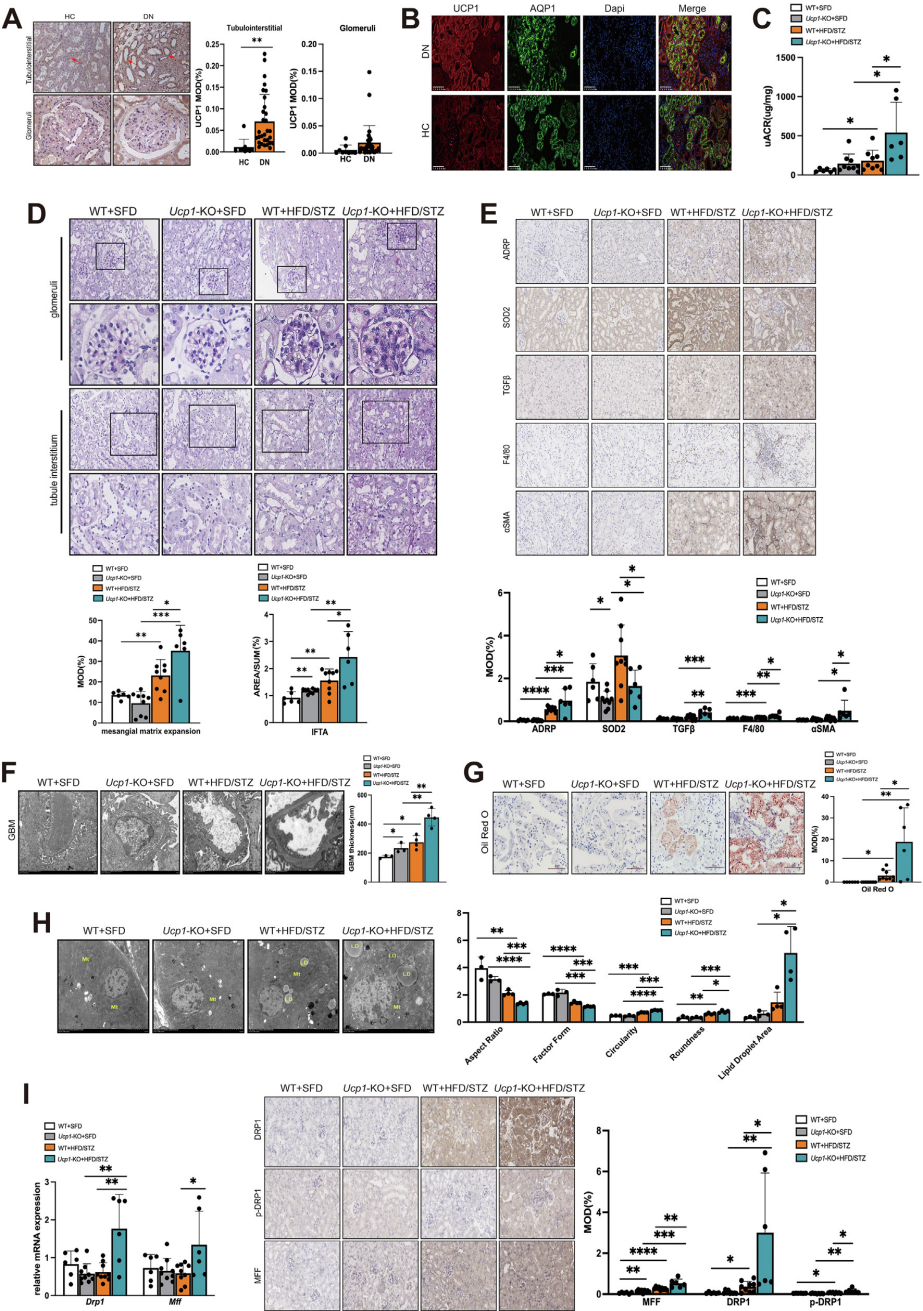
**UCP1 deficiency aggravated renal injuries in diabetes.** (A) Representative IHC images and quantifications of UCP1 expression in the glomeruli and tubulointerstitium of human renal cortical tissues from DN (n = 30) and healthy control (n = 9) in the Chinese cohort (*p* = 0.0080, two-tailed Student’s *t*-test). Scale bar: 50μm. (B) Representative IF images of AQP1 (green) and UCP1 (red) in human renal cortical tissues from DN (n = 30) and healthy control (n = 9) in the Chinese cohort. Scale bar: 100μm. (C) UACR in different groups in mice (n = 6, 9, 9 and 6 respectively, WT+HFD/STZ vs. *Ucp1*-KO+HFD/STZ, *p* = 0.0213, two-way ANOVA followed by Tukey’s post hoc test). (D) Representative PAS photomicrographs and quantifications showing the typical tubulointerstitial (WT+HFD/STZ vs. *Ucp1*-KO+HFD/STZ, *p* = 0.0493) and glomerular structure (WT+HFD/STZ vs. *Ucp1*-KO+HFD/STZ, *p* = 0.0108) in control and diabetic mice (n = 6, 9, 9 and 6 respectively, two-way ANOVA followed by Tukey’s post hoc test). Scale bar:50 μm. (E) Representative IHC images and quantifications of ADRP, SOD2, TGFβ, F4/80 and αSMA in the kidney (n = 6, 9, 9 and 6 respectively, WT+HFD/STZ vs. *Ucp1*-KO+HFD/STZ, *p* = 0.0458, *p* = 0.0431, *p* = 0.0094, *p* = 0.0376 and *p* = 0.0492, two-way ANOVA followed by Tukey’s post hoc test). Scale bar:50 μm. (F) TEM analyses showing GBM thickness in mice (n = 3, 3, 4, 4 respectively, WT+HFD/STZ vs. *Ucp1*-KO+HFD/STZ, *p* = 0.0079, two-way ANOVA followed by Tukey’s post hoc test). Scale bar:2 μm. (G) Representative images and quantitative analysis of Oil Red O staining in mice renal cortex (n = 6, 9, 9 and 6 respectively, WT+HFD/STZ vs. *Ucp1*-KO+HFD/STZ, *p* = 0.0113, two-way ANOVA followed by Tukey’s post hoc test). Scale bar:50 μm. (H) Representative TEM images of proximal tubular cells. Calculation of the form factor (WT+HFD/STZ vs. *Ucp1*-KO+HFD/STZ, *p* = 0.0008), aspect ratio (WT+HFD/STZ vs. *Ucp1*-KO+HFD/STZ, *p* = 0.0003), circularity (WT+HFD/STZ vs. *Ucp1*-KO+HFD/STZ, *p* = 0.0003) and roundness (WT + HFD/STZ vs. *Ucp1*-KO + HFD/STZ, *p* = 0.0339) of mitochondria in diabetic kidney proximal tubular cells from TEM. Lipid droplet percentage (WT+HFD/STZ vs. *Ucp1*-KO+HFD/STZ, *p* = 0.0130) is relative to the total image area (n = 3, 3, 4 and 4 respectively, two-way ANOVA followed by Tukey’s post hoc test). Scale bar:2 μm. (I) mRNA level of *DRP1* and *MFF* in 4 groups (n = 6, 9, 9 and 6 respectively, WT+HFD/STZ vs. *Ucp1*-KO+HFD/STZ, *p* = 0.0077 and *p* = 0.0157, two-way ANOVA followed by Tukey’s post hoc test), representative IHC images and quantification of MFF, DRP1 and p-DRP1 (S616) in the kidney of 4 groups (n = 6, 9, 9 and 6 respectively, WT+HFD/STZ vs. *Ucp1*-KO+HFD/STZ, *p* = 0.0023, *p* = 0.0175 and *p* = 0.0158, two-way ANOVA followed by Tukey’s post hoc test). Scale bar:50 μm. *p < 0.05, **p < 0.01, ***p < 0.001, ****p < 0.0001.

Given the abovementioned importance of UCP1, its specific role in DN warrants further investigation. The expression of UCP1 in kidney specimens in DN patients as well as diabetic mice was detected. Immunohistochemical analysis revealed that UCP1 expression in DN patients (n = 30) was significantly higher compared with healthy controls (n = 9) (Fig. 4A, *p* = 0.0080, two-tailed Student’s *t*-test; Table 1 for general data of the patients). In renal tissues of DN patients, UCP1 was partly colocalized with tubular epithelial cells, podocytes and glomerular endothelial cells as revealed by immunofluorescence colocalization analyses (Fig. S3A). Specifically, compared with healthy controls, UCP1 expression was significantly increased in PTECs (marked as AQP1) in kidney specimens of DN patients in fluorescent staining (Fig. 4B, S3B). Consistently, UCP1 expression was significantly upregulated in kidneys of both *db/db* mice and HFD/STZ-induced diabetic mice compared to their respective littermate controls (Fig. S3C, S3D).

The public available Nephroseq database (http://v5.nephroseq.org) was utilized for further analysis. In the Woroniecka Diabetes TubInt dataset, *UCP1* mRNA levels in the tubulointerstitium positively correlated with GFR in DN patients (n = 22, *p* = 5.97e-4, r = 0.673, Fig. S3E). Additionally, analysis of the Nakagawa CKD cohort revealed that *UCP1* mRNA expression was significantly upregulated in kidney samples from CKD patients (n = 48) compared with non-diseased controls (n = 5, *p* = 1.28e-12, fold change = 4.139, Fig. S3F). These findings suggested a potential relevance of UCP1 in DN, aligning with our own data mentioned above.

Genotype identification and validation of *Ucp1* knockout efficiency at both the mRNA and protein levels were shown in Fig. S3G. WT and *Ucp1^-/-^* mice were treated with HFD/STZ to induce diabetes. Diabetic WT mice developed increased levels of uACR (543.3 ± 385.9 vs. 183.8 ± 131.7 μg/mg, *p* = 0.0213, two-way ANOVA followed by Tukey’s post hoc test), body weight, serum TG and TCHO, which were further aggravated in diabetic *Ucp1^-/-^* mice (Fig. 4C, S3H). PAS analysis suggested that diabetic *Ucp1^-/-^* mice had more severe renal histological damages, including tubulointerstitial injuries and mesangial matrix expansion (Fig. 4D, *p* = 0.0493 and *p* = 0.0108, respectively, two-way ANOVA followed by Tukey’s post hoc test). Compared with diabetic WT mice, there were significantly higher levels of adipose differentiation related protein (ADRP) and profibrotic factors including αSMA and TGFβ, lower levels of superoxide dismutase 2 (SOD2) and more prominent macrophage infiltration (F4/80) in diabetic *Ucp1^-/-^* mice (Fig. 4E, *p* = 0.0458, *p* = 0.0431, *p* = 0.0094, *p* = 0.0376 and *p* = 0.0492, respectively, two-way ANOVA followed by Tukey’s post hoc test). GBM thickness was significantly increased in diabetic *Ucp1^-/-^* mice compared with diabetic WT mice, as assessed by TEM (Fig. 4F, *p* = 0.0079, two-way ANOVA followed by Tukey’s post hoc test). Oil Red O staining revealed an increased lipids deposition in kidneys of diabetic *Ucp1^-/-^* mice compared with diabetic WT mice (Fig. 4G, *p* = 0.0113, two-way ANOVA followed by Tukey’s post hoc test).

TEM analysis showed that diabetic *Ucp1^-/-^* mice had significantly larger lipid droplets in PTECs compared with diabetic WT mice. Furthermore, mitochondrial morphology was more prominently damaged in diabetic *Ucp1^-/-^* mice (Fig. 4H, *p* = 0.0008, *p* = 0.0003, *p* = 0.0003, *p* = 0.0339 and *p* = 0.0130, respectively, two-way ANOVA followed by Tukey’s post hoc test). The levels of mitochondrial fission related proteins including *MFF* and *DRP1* were significantly upregulated in diabetic *Ucp1^-/-^* mice, as revealed by qPCR (*p* = 0.0077, *p* = 0.0157, respectively, two-way ANOVA followed by Tukey’s post hoc test). Consistently, immunohistochemical staining analyses revealed increased expressions of MFF, DRP1 and phosphorylation of DRP1 at Ser616 (p-DRP1 (S616)), a key activation site promoting mitochondrial fission, in diabetic *Ucp1^-/-^* mice compared with diabetic WT mice (Fig. 4I, *p* = 0.0023, *p* = 0.0175 and *p* = 0.0158, respectively, two-way ANOVA followed by Tukey’s post hoc test).

Overall, UCP1 deficiency exacerbated kidney injury and mitochondrial disorders in the context of diabetes.

### UCP1 improved mitochondrial dynamics through the ARX/CRLS1 pathway in diabetes

As described above, UCP1 deficiency disrupted mitochondrial morphology and dynamics in DN. Therefore, the underlying mechanism deserves further exploration.

**Fig. 5:**
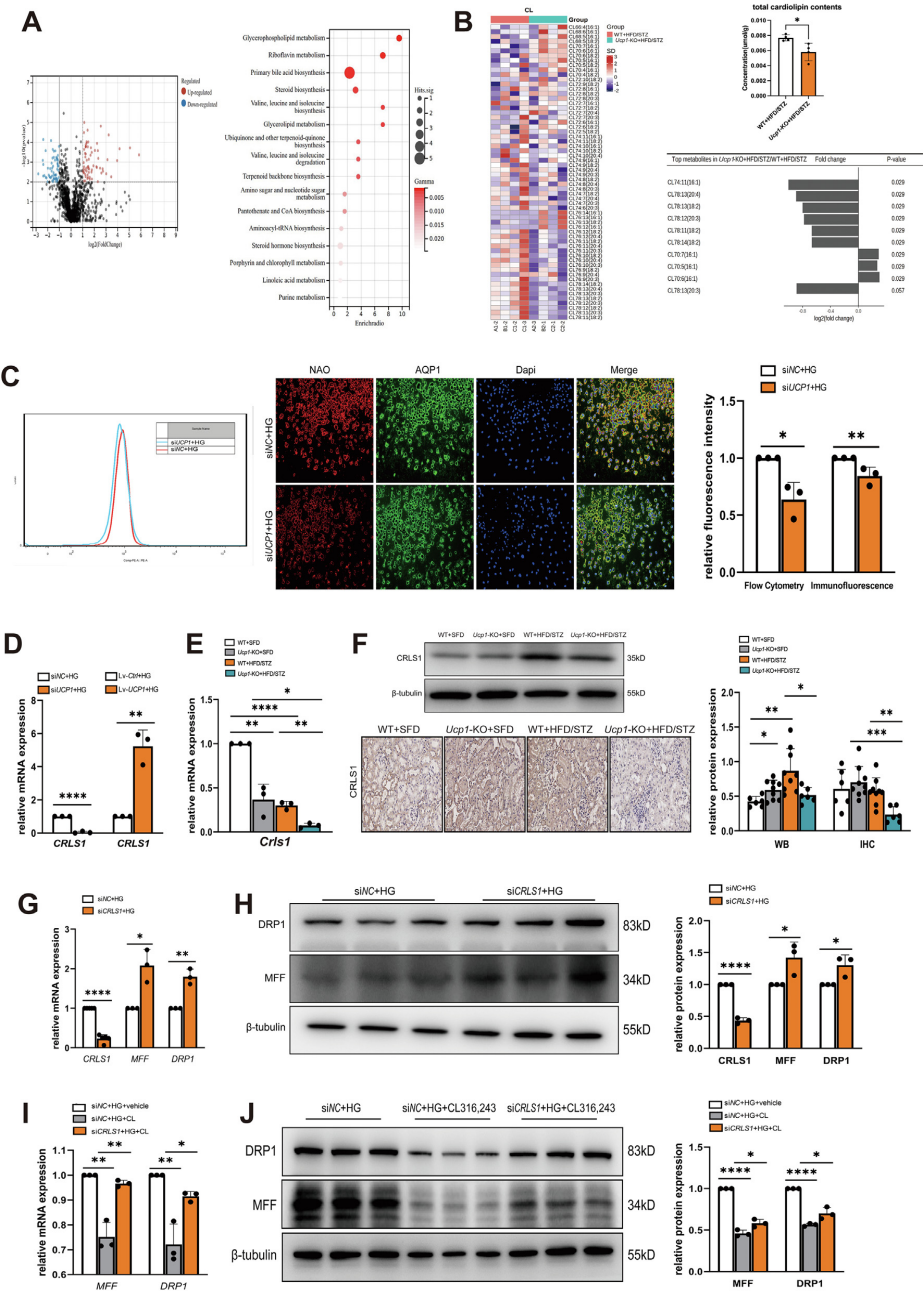
**UCP1 improved mitochondrial dynamics through the ARX/CRLS1 pathway in diabetes.** (A) Pathway enrichment analysis of differentially expressed genes in non-targeted metabolomics showed that the glycerophospholipid metabolism was significantly enriched (n = 7 for diabetic WT group, n = 6 for diabetic *Ucp1^-/-^* group). (B) Lipidomics suggested a significant reduction in cardiolipin contents in diabetic *Ucp1^-/-^* group (n = 4 for diabetic WT group, n = 4 for diabetic *Ucp1^-/-^* group). (C) Representative flow cytometry and IF images and quantifications of cardiolipin probe in HK-2 cells (marked as AQP1) transfected with si*UCP1* under HG conditions (n = 3 per group, si*NC*+HG vs. si*UCP1*+HG, *p* = 0.0139 and *p* = 0.0037, respectively, two-tailed Student’s *t*-test). Scale bar: 100μm. (D) Relative mRNA level of *CRLS1* upon *UCP1* silencing and overexpressing (n = 3 per group, si*NC*+HG vs. si*UCP1*+HG, *p* < 0.0001; Lv-*Ctrl*+HG vs. Lv-*UCP1*+HG, *p* = 0.0017, two-tailed Student’s *t*-test). (E) Relative mRNA level of *CRLS1* in kidneys of 4 groups of mice (n = 3 per group, WT+HFD/STZ vs. *Ucp1*-KO+HFD/STZ, *p* = 0.0015, two-way ANOVA followed by Tukey’s post hoc test). (F) Representative Western blot and IHC images and quantifications of CRLS1 in kidneys of 4 groups of mice (n = 6, 9, 9 and 6 respectively, WT+HFD/STZ vs. *Ucp1*-KO+HFD/STZ, *p* = 0.0415 and *p* = 0.0020, respectively, two-way ANOVA followed by Tukey’s post hoc test). Scale bar: 50μm. (G) Relative mRNA level of *CRLS1*, *MFF* and *DRP1* upon *CRLS1* silencing (n = 3 per group, si*NC*+HG vs. si*CRLS1*+HG, *p* < 0.0001, *p* = 0.0104 and *p* = 0.0021, respectively, two-tailed Student’s *t*-test). (H) A representative Western blot (n = 3 blots in total) and quantification of CRLS1 upon *CRLS1* silencing (si*NC*+HG vs. si*CRLS1*+HG, *p* < 0.0001, *p* = 0.0409 and *p* = 0.0318, respectively, two-tailed Student’s *t*-test). (I) Relative mRNA level of *MFF* and *DRP1* among 3 groups (n = 3 per group, si*NC*+HG+CL vs. si*CRLS1*+HG+CL, *p* = 0.0035 and *p* = 0.0165, one-way ANOVA followed by Tukey’s post hoc test). (J) A representative Western blot (n = 3 blots in total) and quantifications of MFF and DRP1 among 3 groups (si*NC*+HG+CL vs. si*CRLS1*+HG+CL, *p* = 0.0262 and *p* = 0.0325, respectively, one-way ANOVA followed by Tukey’s post hoc test). *p < 0.05, **p < 0.01, ***p < 0.001, ****p < 0.0001.

Untargeted metabolomics profiling revealed 68 upregulated and 60 downregulated differential metabolites in kidney tissues of diabetic *Ucp1^-/-^* mice compared to diabetic WT mice. Pathway enrichment analysis suggested that glycerophospholipid metabolism was the most significantly enriched one (Fig. 5A). Among glycerophospholipids, cardiolipin (CL), a signature one found almost exclusively on the inner mitochondrial membrane [32,33], maintains mitochondrial homeostasis by regulating mitochondrial fusion and fission [34]. It was reported that UCP1 not only increased CL contents [35,36], but also bound CL stably [37]. Compared with diabetic WT controls, total cardiolipin contents in the renal cortex were reduced in diabetic *Ucp1^-/-^* mice. Notably, it was found that specific cardiolipin species, including CL74:11(16:1), CL78:13(20:4), CL78:13(18:2), CL78:12(20:3), CL78:11(18:2), CL78:14(18:2) and CL78:13(20:3), were significantly decreased in renal cortex of diabetic *Ucp1^-/-^* mice compared with diabetic WT controls (Fig. 5B).

*In vitro*, it was found that cardiolipin levels were decreased upon *UCP1* silencing in HK-2 cells under HG conditions by flow cytometry and IF assays (Fig. 5C, *p* = 0.0139 and *p* = 0.0037, respectively, two-tailed Student’s *t*-test), which was consistent with the above results of animal study. CRLS1, encoded by *CRLS1*, a key enzyme in cardiolipin biosynthesis [38], were significantly reduced upon *UCP1* silencing and increased upon *UCP1* overexpressing in HK-2 cells under HG conditions (Fig. 5D, si*NC*+HG vs. si*UCP1*+HG, *p* < 0.0001; Lv-*Ctrl*+HG vs. Lv-*UCP1*+HG, *p* = 0.0017, two-tailed Student’s *t*-test). Consistently, CRLS1 expression was downregulated at both the mRNA and protein levels in diabetic *Ucp1^-/-^* mice compared to diabetic WT mice (Fig. 5E, *p* = 0.0015, two-way ANOVA followed by Tukey’s post hoc test; 5F, *p* = 0.0415 and *p* = 0.0020, respectively, two-way ANOVA followed by Tukey’s post hoc test). Next, under HG conditions, it was found that in si*CRLS1*-treated HK-2 cells, mitochondrial fission was enhanced, as evidenced by increased levels of DRP1 and MFF at both the mRNA and protein levels, compared with the si*NC* group (Fig. 5G, *p* < 0.0001, *p* = 0.0104 and *p* = 0.0021, respectively, two-tailed Student’s *t*-test; 5H, *p* < 0.0001, *p* = 0.0409 and *p* = 0.0318, respectively, two-tailed Student’s *t*-test). Similar results were observed in primary MPTECs under HG conditions (Fig. S4A, S4B). Furthermore, 3,5-dimethylpyrazole-pyridine-chlorodinitrobenzene conjugate (PPCD) was employed to inhibit cardiolipin biosynthesis in both HK-2 cells and primary MPTECs. NAO probe staining showed that the content of cardiolipin decreased upon PPCD treatment in both cell types (Fig. S4C, S4D). Western blot and qPCR analysis revealed that PPCD treatment significantly increased the expression of MFF and p-DRP1 (S616) compared with vehicle treatment (Fig. S4E-S4H). Moreover, mitochondrial morphology analysis using MitoTracker staining demonstrated that PPCD treatment resulted in a marked decrease in mitochondria, accompanied by disrupted mitochondrial network in HK-2 and primary MPTECs (Fig. S4I, S4J). Under HG conditions, CL316243 treatment reduced the expression of DRP1 and MFF. However, this downregulation was significantly attenuated in *CRLS1*-silenced HK-2 cells at both the mRNA and protein levels (Fig. 5I, *p* = 0.0035 and *p* = 0.0165, one-way ANOVA followed by Tukey’s post hoc test; 5J, *p* = 0.0262 and *p* = 0.0325, one-way ANOVA followed by Tukey’s post hoc test). Moreover, the beneficial effects of CL316243, including the reduction of HG-induced ROS accumulation and lipid peroxidation, were also abrogated by *CRLS1* knockdown (Fig. S4K). Given that UCP1 deficiency could downregulate *CRLS1* at the mRNA level, transcription factors of *CRLS1* were predicted, and three were selected through *in vivo* and *in vitro* validation (Fig. S4L). ChIP assay revealed that aristaless-related homeobox (ARX), but not other candidate transcription factors, specifically bound to the predicted promoter region of *CRLS1*. Furthermore, dual-luciferase reporter assays revealed that ARX-dependent promoter activation was significantly attenuated under *UCP1*-deficient conditions, indicating that UCP1 modulated ARX transcriptional activity (Fig. S4M). As shown in Fig. S4N-4Q, *UCP1*/*Ucp1* silencing significantly reduced, while *UCP1*/*Ucp1* overexpression significantly increased, ARX expression at both the mRNA and protein levels in both HK-2 and primary MPTECs. These results indicated that UCP1 positively regulated ARX expression. Moreover, the subcellular localization of ARX was examined and it was found that *UCP1/Ucp1* knockdown diminished, whereas *UCP1/Ucp1* overexpression enhanced, ARX nuclear translocation, as revealed by immunofluorescence staining in both cell types (Fig. S4R). In line with these, *ARX* knockdown under HG conditions significantly reduced CRLS1 protein expression compared with si*NC* controls (Fig. S4S). Besides, in *UCP1*-KO HK-2 cells exposed to HG, *ARX* overexpression restored CRLS1 protein expression, cardiolipin contents, and improved mitochondrial morphology (Fig. S4T), indicating that ARX mediated UCP1-dependent CRLS1 regulation.

*In vivo*, AAV9 overexpressing *Crls1* or *Ctrl* and carrying KSPC promoter was delivered in HFD/STZ-induced diabetic *Ucp1^-/-^* mice by tail vein and local renal cortex injection (tail-AAV9-*Crls1*/*Ctrl*, renal-AAV9-*Crls1*/*Ctrl*) (Fig. S5A). Compared with diabetic *Ucp1^-/-^* tail-AAV9-*Ctrl* group, serum TG, TCHO and uACR were significantly decreased in diabetic *Ucp1^-/-^* tail-AAV9-*Crls1* group (Fig. S5B). Compared with diabetic *Ucp1^-/-^* tail-AAV9-*Ctrl* group, overexpression of *Crls1* by tail vein injection significantly reduced IFTA score and the degree of mesangial matrix expansion in renal histology (Fig. S5C). GBM thickness was significantly decreased, and mitochondrial morphologic damages were improved in diabetic *Ucp1^-/-^* tail-AAV9-*Crls1* group (Fig. S5D). Overexpression of *Crls1* by tail vein injection reversed the disorder of mitochondrial fission induced by *Ucp1* knockout (Fig. S5E). In addition, compared with diabetic *Ucp1^-/-^* renal-AAV9-*Ctrl* group, blood glucose was significantly decreased in diabetic *Ucp1^-/-^* renal-AAV9-*Crls1* group (Fig. S5F), and there was also a borderline improvement in uACR (86.10 ± 40.73 vs. 210.2 ± 146.2 μg/mg, *p* = 0.0515) (Fig. S5G). PAS, TEM and immunohistochemical staining results showed that in diabetic *Ucp1^-/-^* renal-AAV9-*Crls1* mice, IFTA score, the degree of mesangial matrix expansion and GBM thickness were significantly decreased, and mitochondrial morphologic damages were evidently alleviated, compared with diabetic *Ucp1^-/-^* renal-AAV9-*Ctrl* group (Fig. S5H, S5I, S5J). Thus, renal overexpression of *Crls1* ameliorated the renal phenotype and mitochondrial fission of diabetic *Ucp1^-/-^* mice.

Collectively, these results suggested that UCP1 improved mitochondrial dynamics through the ARX/CRLS1 pathway in diabetes.

### Upregulating or activating UCP1 improved mitochondrial homeostasis and attenuated established DN

**Fig. 6:**
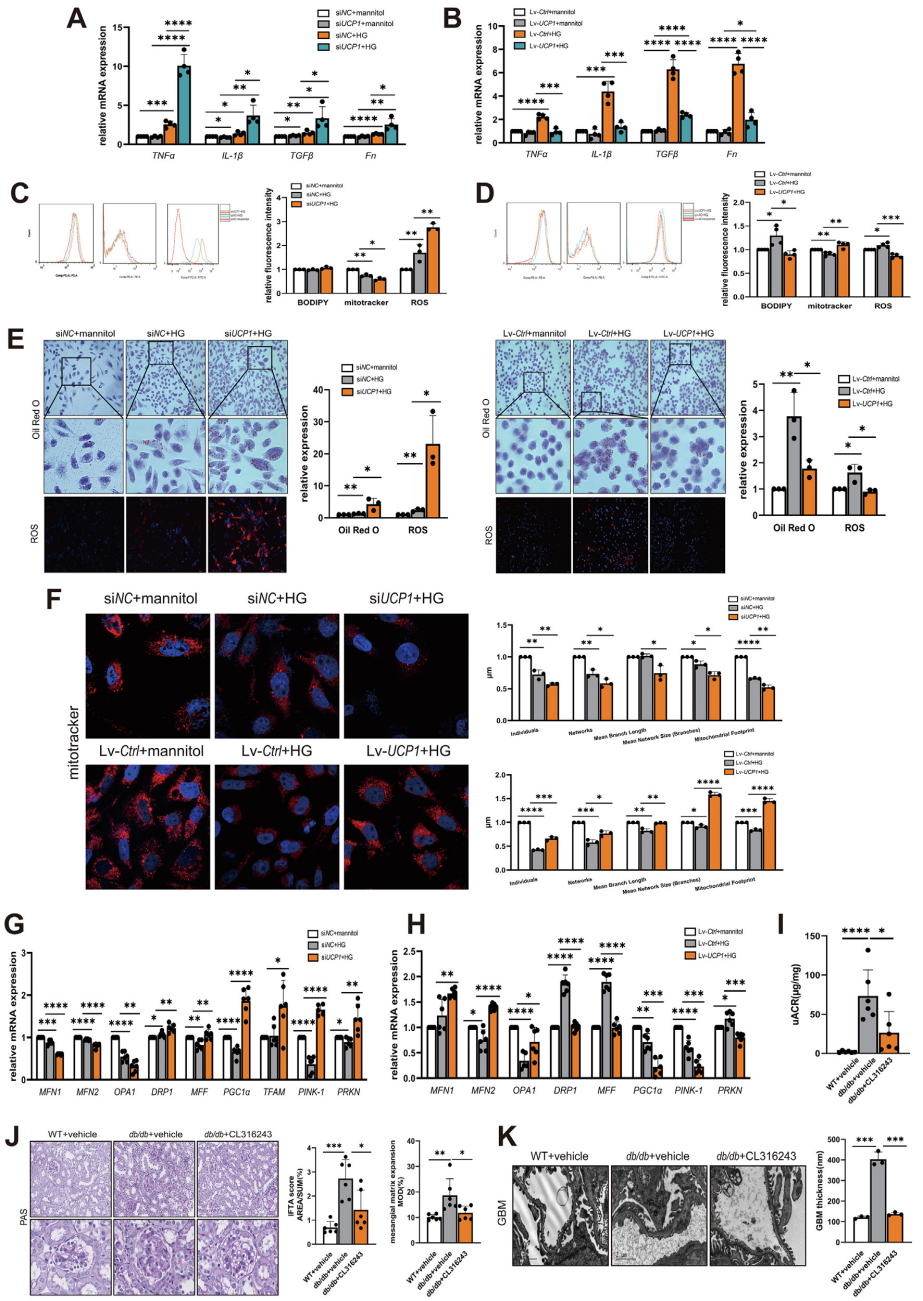
**Upregulating or activating UCP1 improved mitochondrial homeostasis and attenuated established DN.** (A) Relative mRNA levels of *TNFα*, *IL-1β*, *Fn* and *TGFβ* upon *UCP1* silencing (n = 4 per group, si*NC*+HG vs. si*UCP1*+HG, *p* < 0.0001, *p* = 0.0133, *p* = 0.0172 and *p* = 0.0456, respectively, two-way ANOVA followed by Tukey’s post hoc test). (B) Relative mRNA levels of *TNFα*, *IL-1β*, *Fn* and *TGFβ* upon *UCP1* overexpressing (n = 4 per group, Lv-*Ctrl*+HG vs. Lv-*UCP1*+HG, *p* = 0.0003, *p* = 0.0007, *p* = 0.0001 and *p* < 0.0001, respectively, two-way ANOVA followed by Tukey’s post hoc test). (C) Representative images and quantifications of BODIPY, ROS (si*NC*+HG vs. si*UCP1*+HG, *p* = 0.0081) and mitotracker (si*NC*+HG vs. si*UCP1*+HG, *p* = 0.0222) by flow cytometry upon *UCP1* silencing (n = 3 per group, one-way ANOVA followed by Tukey’s post hoc test). (D) Representative images and quantifications of BODIPY (Lv-*Ctrl*+HG vs. Lv-*UCP1*+HG, *p* = 0.0113), ROS (Lv-*Ctrl*+HG vs. Lv-*UCP1*+HG, *p* = 0.0006) and mitotracker (Lv-*Ctrl*+HG vs. Lv-*UCP1*+HG, *p* = 0.0021) by flow cytometry upon *UCP1* overexpressing (n = 3 per group, one-way ANOVA followed by Tukey’s post hoc test). (E) Representative images and quantifications of Oil Red O (si*NC*+HG vs. si*UCP1*+HG, *p* = 0.0365; Lv-*Ctrl*+HG vs. Lv-*UCP1*+HG, *p* = 0.0445) and ROS (si*NC*+HG vs. si*UCP1*+HG, *p* = 0.0156; Lv-*Ctrl*+HG vs. Lv-*UCP1*+HG, *p* = 0.0201) staining in HK-2 cells upon *UCP1* silencing and overexpressing (n = 3 per group, one-way ANOVA followed by Tukey’s post hoc test). Scale bar: 50μm. (F) Representative photographs of mitochondria labeled with mitotracker. Quantification of individual mitochondria, mitochondrial networks, footprints, and mean branches per network by Mitochondrial Network Analysis toolset in *UCP1* silencing (si*NC*+HG vs. si*UCP1*+HG, *p* = 0.0239, *p* = 0.0468, *p* = 0.0197, *p* = 0.0200 and *p* = 0.0035, respectively) and overexpressing (Lv-*Ctrl*+HG vs. Lv-*UCP1*+HG, *p* = 0.0006, *p* = 0.0165, *p* = 0.0033, *p* < 0.0001 and *p* < 0.0001, respectively) HK-2 cells labeled with mitotracker (n = 3 per group, one-way ANOVA followed by Tukey’s post hoc test). Scale bar: 5 μm. (G) Relative mRNA level of markers of mitochondrial homeostasis upon *UCP1* silencing (n = 6 per group, si*NC*+HG vs. si*UCP1*+HG, *p* < 0.0001, *p* = 0.0001, *p* = 0.0086, *p* = 0.0082, *p* = 0.0034, *p* < 0.0001, *p* = 0.0252, *p* < 0.0001 and *p* = 0.0026, respectively, one-way ANOVA followed by Tukey’s post hoc test). (H) Relative mRNA level of markers of mitochondrial homeostasis upon *UCP1* overexpressing (n = 6 per group, Lv-*Ctrl*+HG vs. Lv-*UCP1*+HG, *p* = 0.0090, *p* < 0.0001, *p* = 0.0299, *p* < 0.0001, *p* < 0.0001, *p* = 0.0005, *p* = 0.0010 and *p* = 0.0003, respectively, one-way ANOVA followed by Tukey’s post hoc test). (I) uACR in different groups in mice (n = 6 per group, *db*/*db*+vehicle vs. *db*/*db*+CL316243, *p* = 0.0130, one-way ANOVA followed by Tukey’s post hoc test). (J) Representative PAS photomicrographs and quantifications showing typical tubulointerstitial (*db*/*db*+vehicle vs. *db*/*db*+CL316243, *p* = 0.0198) and glomerular structure (*db*/*db*+vehicle vs. *db*/*db*+CL316243, *p* = 0.0380) in mice (n = 6 per group, one-way ANOVA followed by Tukey’s post hoc test). Scale bar:50 μm. (K) TEM analyses showing GBM thickness in mice (n = 3 per group, *db*/*db*+vehicle vs. *db*/*db*+CL316243, *p* = 0.0002, one-way ANOVA followed by Tukey’s post hoc test). Scale bar:2 μm. *p < 0.05, **p < 0.01, ***p < 0.001, ****p < 0.0001.

Stable *UCP1* overexpression and silencing in HK-2 cells were established for our following experiments (Fig. S6A, S6B). Under HG conditions, the expression of *TNFα*, *IL-1β*, *TGFβ* and *Fn* was significantly upregulated upon *UCP1* silencing compared with the si*NC* group, and was significantly downregulated upon *UCP1* overexpressing compared with the Lv-*Ctrl* group (Fig. 6A, *p* < 0.0001, *p* = 0.0133, *p* = 0.0172 and *p* = 0.0456, respectively, two-way ANOVA followed by Tukey’s post hoc test; 6B, *p* = 0.0003, *p* = 0.0007, *p* = 0.0001 and *p* < 0.0001, respectively, two-way ANOVA followed by Tukey’s post hoc test). The number of mitochondria was reduced prominently accompanied by accumulation of ROS, lipid peroxidation and lipid droplets under HG conditions. These metabolic disorders induced by HG were further aggravated when *UCP1* was silenced, and attenuated when *UCP1* was overexpressed (Fig. 6C, 6D, 6E, one-way ANOVA followed by Tukey’s post hoc test). In addition, several quantitative indicators of mitochondrial morphology were all significantly decreased under HG conditions compared with mannitol controls, including mitochondrial individuals, networks, mean branch length, mean network size and footprint. Such disorders were aggravated upon *UCP1* silencing and alleviated upon *UCP1* overexpressing (Fig. 6F, si*NC*+HG vs. si*UCP1*+HG, *p* = 0.0239, *p* = 0.0468, *p* = 0.0197, *p* = 0.0200 and *p* = 0.0035; Lv-*Ctrl*+HG vs. Lv-*UCP1*+HG, *p* = 0.0006, *p* = 0.0165, *p* = 0.0033, *p* < 0.0001 and *p* < 0.0001, one-way ANOVA followed by Tukey’s post hoc test). Compared with control groups, at the mRNA level, mitochondrial fusion indicators including *MFN1*, *MFN2* and *OPA1* were significantly decreased, while mitochondrial fission indicators including *MFF* and *DRP1* were significantly increased, accompanied by increased mitochondrial biogenesis (indicated by *PGC1α* and *TFAM*) and mitophagy (indicated by PTEN-induced putative kinase 1, *PINK1* and parkin RBR E3 ubiquitin protein ligase, *PRKN*) in *UCP1* silencing HK-2 cells treated with HG (Fig. 6G, *p* < 0.0001, *p* = 0.0001, *p* = 0.0086, *p* = 0.0082, *p* = 0.0034, *p* < 0.0001, *p* = 0.0252, *p* < 0.0001 and *p* = 0.0026, respectively, one-way ANOVA followed by Tukey’s post hoc test). Correspondingly, the above markers showed opposite results upon *UCP1* overexpressing (Fig. 6H, *p* = 0.0090, *p* < 0.0001, *p* = 0.0299, *p* < 0.0001, *p* < 0.0001, *p* = 0.0005, *p* = 0.0010 and *p* = 0.0003, respectively, one-way ANOVA followed by Tukey’s post hoc test). In addition, the protein levels of MFF and p-DRP1 (S616) were significantly increased upon *Ucp1* silencing, while decreased upon *Ucp1* overexpressing in primary MPTECs under HG conditions (Fig. S6C, S6D). Taken together, UCP1 could maintain mitochondrial homeostasis under diabetes-mimicking conditions *in vitro*.

To determine whether the protective effects of CL316243 are dependent on UCP1, *in vitro* experiments using both HK-2 cells and primary MPTECs were performed. Under HG conditions, CL316243 treatment significantly attenuated the expression of proinflammatory and profibrotic factors, including *TNFα*, *IL-1β*, *TGFβ*, *FN*, *αSMA*, and *F4/80,* as well as tubular injury markers including *KIM-1/Kim-1* and *NGAL/Ngal*. In addition, CL316243 attenuated the increase in *MFF/Mff* and *DRP1/Drp1*, indicating a restoration of the balance of mitochondrial dynamics. However, these beneficial effects were significantly abolished upon *UCP1*/*Ucp1* knockdown in both cell types (Fig. S6E, S6F).

We next evaluated whether CL316243 could ameliorate established DN *in vivo*. CL316243-treated *db/db* mice manifested significantly decreased uACR (26.27 ± 27.28 vs. 73.37 ± 33.19 μg/mg, *p* = 0.013, one-way ANOVA followed by Tukey’s post hoc test) compared with the vehicle-treated mice (Fig. 6I). Mesangial matrix expansion and IFTA score were significantly improved upon CL316243 treatment (Fig. 6J, *p* = 0.0198 and *p* = 0.0380, one-way ANOVA followed by Tukey’s post hoc test). TEM analysis showed that CL316243 treatment alleviated GBM thickening compared with vehicle-treatment (Fig. 6K, *p* = 0.0002, one-way ANOVA followed by Tukey’s post hoc test). Therefore, CL316243 could attenuate established DN in *db/db* mice.

## Discussion

Our previous studies demonstrated that ANXA1 alleviated lipid metabolism disorders by regulating mitochondrial homeostasis in DN[11,12], but the specific mechanism remains unclear. In the current study, *Ucp1* was identified as the most significantly downregulated FAO metabolism and mitochondria-associated gene in the renal cortex of diabetic *Anxa1*-KO mice compared with diabetic WT mice, thereby prompting us to focus its role on mitochondrial homeostasis and DN pathophysiology. This study aimed to explore the underlying mechanisms by which ANXA1 regulated mitochondrial homeostasis and to evaluate the potential as a therapeutic target for mitochondrial-based treatments for DN.

One of the key findings of this study was the identification of UCP1 as the central mediator of the nephroprotective effects of ANXA1 in DN. Previous studies have demonstrated that ANXA1 protected diabetic kidney by restoring Akt and MAPK signaling pathway and reducing oxidative stress in type 1 diabetes [7], and by inhibiting RhoA to alleviate inflammation, enhance insulin sensitivity and improve renal function in type 2 diabetes [8]. Different from these previous studies, our current study discovered a novel and well-supported pathway based on comprehensive transcriptomic profiling, thereby identifying a previously unrecognized mechanism by which ANXA1 exerts its nephroprotective role in DN. Besides, recent studies suggested that ANXA1 regulated mitochondrial function in cancer and cardiovascular disease [9,10], and our findings extended these roles to the field of DN. While UCP1 is traditionally recognized for its role in adipose tissue thermogenesis, recent studies have highlighted its broader functions in regulating oxidative phosphorylation efficiency and mitochondrial redox homeostasis [39]. Specifically, UCP1-mediated uncoupling may reduce mitochondrial membrane potential, thereby attenuating ROS production under hyperglycemic conditions, which was reported as a protective mechanism in non-renal tissues [40,41]. Since mitochondrial dysfunction and oxidative stress play a central role in the pathogenesis of DN, such controlled uncoupling could serve to maintain mitochondrial homeostasis and prevent mitochondria-triggered inflammation and apoptosis. Thus, upregulation of UCP1 mediated by ANXA1 probably represents a compensatory adaptation to mitigate mitochondrial stress and injury. Mechanistically, it was demonstrated in our study that ANXA1 stabilized GATA3, a process that may involve thioredoxin-mediated regulation, and it was reasonable to propose the ASK1/MAPK pathway as a putative link, as supported by the previous study [42]. Although a transcriptional mechanism by which ANXA1 regulated UCP1 was elucidated in our study, whether there are other mechanisms such as post-translational modifications, structural alterations and epigenetic regulation of UCP1, which modulate its uncoupling activity in brown adipose tissues [43,44], warrants further investigation in diabetic kidney.

After identifying UCP1 as a key downstream effector of ANXA1, the role of UCP1 in mitochondrial regulation in DN was further investigated. To this end, another important finding of the current study was to unravel the important role of UCP1 in the pathogenesis of DN, and its regulation of cardiolipin biosynthesis through the ARX/CRLS1 pathway to maintain mitochondrial homeostasis. The pathophysiological role of UCP1 has been largely overlooked in DN previously. Danhong injection was reported to upregulate UCP1 and improve renal phenotype in diabetic rats; however, the specific role of UCP1 in this process has not been investigated [45]. Thus, our study addressed this gap by elucidating the functional involvement of UCP1 in DN. Although UCP1 was traditionally not recognized as a direct regulator of cardiolipin metabolism, emerging evidence supported that mitochondrial uncoupling proteins, including UCP1, could influence mitochondrial membrane composition and cristae structure [46]. In addition, a recent study reported a tight and direct physical association between UCP1 and cardiolipin, suggesting that cardiolipin acts as a structural co-factor that promotes UCP1 localization and stability within the inner mitochondrial membrane [37]. It was found in our study that UCP1 deficiency aggravated diabetes-induced kidney injury, led to decreased renal cardiolipin contents and enhanced mitochondrial fission, which was consistent with previous findings that cardiolipin depletion exacerbated mitochondrial membrane permeability and fission [47,48]. Furthermore, our findings established a mechanistic axis wherein UCP1 positively regulated ARX expression, which in turn transcriptionally activated CRLS1, thereby enhancing cardiolipin biosynthesis and improving mitochondrial dynamics. Given the lack of direct evidence linking ARX to CRLS1 previously, our findings represented a novel regulatory axis in PTECs in the context of diabetes. In this light, the UCP1/ARX/CRLS1 pathway may serve as a unique mechanism to support mitochondrial adaptation in diabetic stress. Interestingly, in addition to mitochondrial fission and fusion in the mitochondrial quality control system, the levels of mitochondrial biogenesis and mitophagy were elevated in UCP1-deficient HK-2 cells under HG conditions, likely as compensatory responses to remove damaged mitochondria. However, whether these compensatory responses were adaptive or maladaptive in chronic diabetic conditions remains to be determined. Collectively, these findings further highlighted the protective role of UCP1 in maintaining mitochondrial homeostasis in DN.

Given its important role in maintaining mitochondrial homeostasis, UCP1 was further explored as a therapeutic target for DN in the current study. The application of CL316243 evidently attenuated established DN in *db*/*db* mice, further supporting the feasibility of pharmacologically modulating UCP1 in DN treatment. Previous studies reported that chemical uncoupling agents, such as 2,4-dinitrophenol (DNP) and valinomycin, were unsuitable for clinical use due to severe adverse effects [49]. UCP1-specific modulators may offer a potentially safer alternative for DN treatment. Although our preliminary *in vitro* experiments suggested that the protective effects of CL316243 were at least partially UCP1-dependent, its specificity in targeting UCP1 remains to be further validated. In addition, further investigation is needed to evaluate its safety, particularly concerning the risk of cellular energy depletion resulting from excessive UCP1 activation.

Despite these findings, there were certain limitations in this study. First, *Ucp1* global knockout mice rather than PTECs-specific knockout ones were employed in our study, making it difficult to exclude potential contributions of UCP1 from other tissues or cells to the observed kidney phenotype. Second, whether ANXA1 regulates mitochondrial homeostasis in DN through additional pathways, and how these pathways interact with UCP1, remains to be fully elucidated. Third, due to technical constraints, direct TEM immunogold localization of cardiolipin in renal tissue could not be performed at this stage. Finally, the uncertain specificity of CL316243 in targeting UCP1 for renal protection in DN represented another limitation of this study, which needs further validation.

## Conclusion

Taken together, this study identified UCP1 as a key mediator of the nephroprotective effects of ANXA1 in DN by regulating mitochondrial homeostasis. Mechanistically, ANXA1 stabilized GATA3, thereby enhancing the transcription of *UCP1*. UCP1, in turn, increased cardiolipin contents through the ARX/CRLS1 pathway, which reduced excessive mitochondrial fission and maintained mitochondrial homeostasis in renal tubular epithelial cells under diabetic conditions. Our study has two novelties: first, the elucidation of the molecular mechanism by which ANXA1 regulates mitochondrial homeostasis in DN; and second, the identification of targeting UCP1 as a potential therapeutic strategy for DN. These findings effectively addressed the initial research objective of our study.

**Data availability**

The untargeted metabolomics and cardiolipin content analysis data are available in Supplementary File 1. Additional data in this article, such as reagents, resources, software, other useful materials and methods details are available in Supplementary File 2 and Supplementary Methods. Data in this study are available for access on Zenodo.org (doi.org/10.5281/zenodo.10435506) upon request.

Supplemental information

The Excel file was related to Supplementary File 1 and raw data and statistics.

KEY RESOURCES TABLE and Fig. S1-6 with legends in Supplementary File 2.

**Compliance with Ethics Requirements**

All Institutional and National Guidelines for the care and use of animals (fisheries) were followed.

**Compliance with Ethics Requirements**

All procedures followed were in accordance with the ethical standards of the responsible committee on human experimentation (institutional and national) and with the Helsinki Declaration of 1975, as revised in 2008 (5). Informed consent was obtained from all patients for being included in the study

**Ethics approval and consent to participate**

This study was approved by the Ethics Committee of Peking University First Hospital, with the approval number of 2023[135–002]. Written informed consent for tissue donation, which clearly stated the purpose of our study, were obtained from all of the participates.

## CRediT authorship contribution statement

**Zi-Han Li:** Writing – original draft, Conceptualization, Data curation, Formal analysis, Investigation, Software. **Lu Fang:** Methodology, Project administration, Resources, Validation. **Liang Wu:** Conceptualization, Funding acquisition, Methodology, Validation. **Dong-Yuan Chang:** Methodology, Project administration, Resources. **Manyuan Dong:** Methodology. **Liang Ji:** Methodology. **Qi Zhang:** Methodology. **Ming-Hui Zhao:** Funding acquisition, Project administration, Resources, Writing – review & editing. **Sydney C.W. Tang:** Project administration, Resources, Writing – review & editing. **Lemin Zheng:** Funding acquisition, Project administration, Resources, Writing – review & editing, Validation. **Min Chen:** Funding acquisition, Project administration, Conceptualization, Writing – review & editing, Supervision, Validation.

## Declaration of competing interest

The authors declare that they have no known competing financial interests or personal relationships that could have appeared to influence the work reported in this paper.
