# Supplementary material for: ANXA1 improves mitochondrial homeostasis through uncoupling protein 1 in diabetic nephropathy
Source: J Adv Res. 2025 Nov 22;86:689–708. doi: 10.1016/j.jare.2025.11.039 (PMC13453871; doi:10.1016/j.jare.2025.11.039)
Supplement: Supplementary file 1 [file mmc2.pdf]

Supplementary Figures

Figure S1

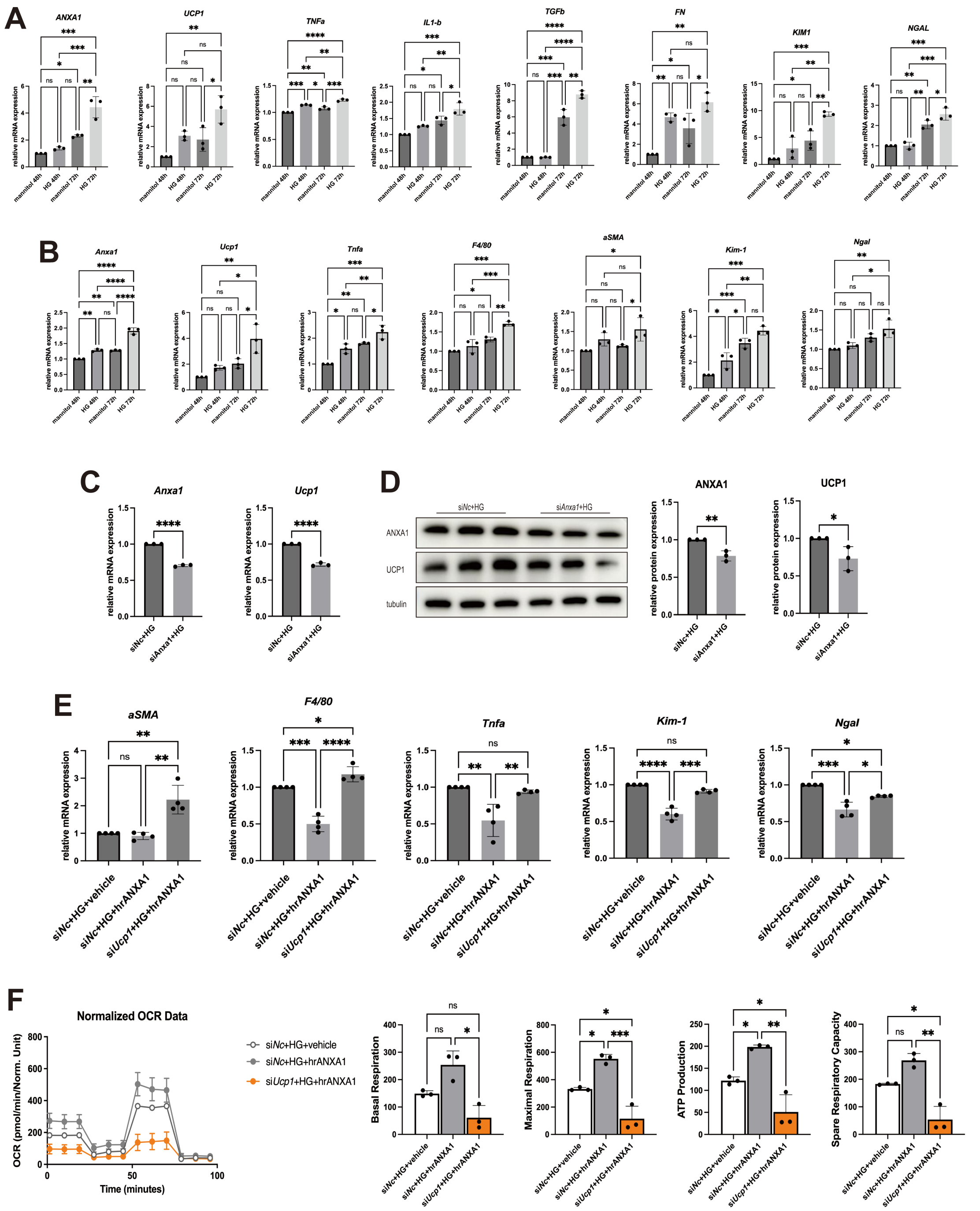

Figure S1. (A) Relative mRNA levels of *ANXA1*, *UCP1*, *TNF-α*, *IL-1β*, *TGFβ*, *FN*, *KIM-1*, *NGAL* in HK-2 cells under different times of mannitol or HG treatment (n = 3 per group, two-way ANOVA followed by Tukey's post hoc test). (B) Relative mRNA

levels of *Anxa1*, *Ucp1*, *Tnf-α*, *F4/80*, *αSMA*, *Kim-1*, *Ngal* in primary murine PTECs under different times of mannitol or HG treatment (n = 3 per group, two-way ANOVA followed by Tukey's post hoc test). (C-D) Relative mRNA and protein levels of ANXA1 and UCP1 upon *Anxa1* silencing in primary murine PTECs under HG condition, with corresponding representative blot images (n = 3 per group, two-tailed Student's *t*-test). (E) Relative mRNA levels of *Tnf-α*, *F4/80*, *αSMA*, *Kim-1*, *Ngal* in primary murine PTECs in three groups (n = 3 per group, two-way ANOVA followed by Tukey's post hoc test). (F) OCR in primary murine PTECs. si*Ucp1* and si*Nc* cells received hrANXA1 treatment exposed to HG for 72 h (n=3 per group). *p<0.05, **p<0.01, ***p<0.001, ****p<0.0001.

Figure S2

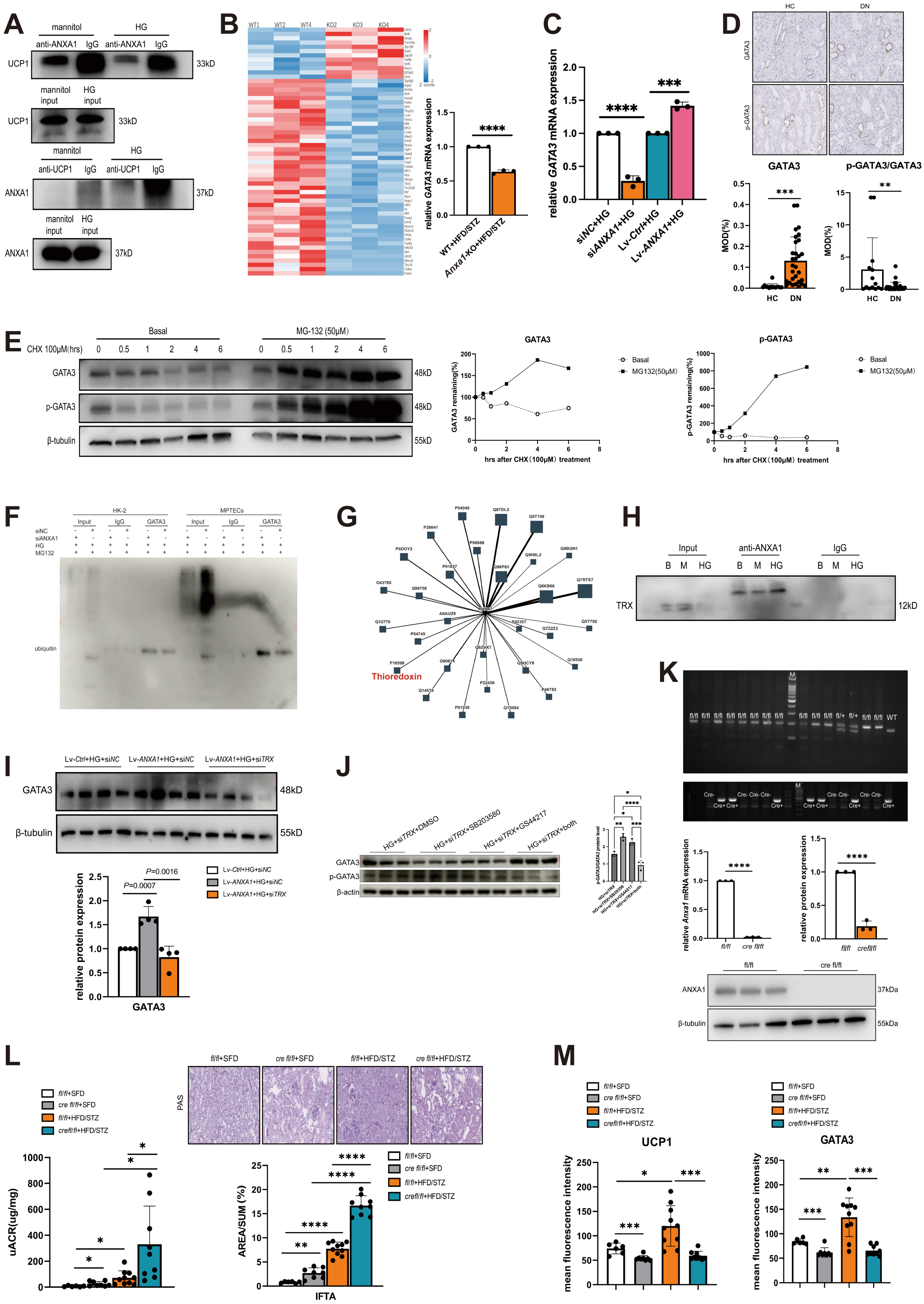

Figure S2. (A) Forward and reverse IP images showing the binding of ANXA1 with UCP1. (B) Relative mRNA level of *GATA3* in diabetic WT and diabetic $Anxa1^{-/-}$ mice

(n = 3 per group, two-tailed Student's *t*-test). (C) Relative mRNA level of *GATA3* in HK-2 cells transfected with si*ANXA1* and Lv-*ANXA1*(n = 3 per group, two-tailed Student's *t*-test). (D) Representative IHC images and quantifications of GATA3 and pGATA3/GATA3 expression in human renal cortical tissues from DN (n = 30) and control (n = 14) in the Chinese cohort (two-tailed Student's *t*-test). Scale bar: 50μm. (E) HK-2 cells were treated with cycloheximide (CHX) at 100 μM final concentration with/ without MG-132 at 50 μM as indicated, protein cell lysates were prepared and GATA3, pGATA3 expressions were measured by WB. (F) A representative IP images showing the binding of GATA3 and ubiquitin in HK-2 cells and primary murine PTECs upon *ANXA1*/*Anxa1* silencing under HG and MG132 condition. (G) Interaction network of ANXA1 and associated proteins identified by mass spectrometry. ANXA1 is shown as the central grey node. Node size reflects the protein interaction score, while color intensity indicates sequence coverage. Thioredoxin (TRX, accession P10599) is specifically highlighted in red to indicate its interaction with ANXA1. (H) A representative IP images showing the binding of ANXA1 and thioredoxin. (I) A representative western blot (n = 4 blots in total) and quantifications of GATA3 among 3 groups (one-way ANOVA followed by Tukey's post hoc test). (J) A representative western blot (n = 3 blots in total) and quantifications of p-GATA3/GATA3 among 4 groups (one-way ANOVA followed by Tukey's post hoc test). (K) Representative genotype identification and knockout efficiency validation in both mRNA and protein levels (n = 3 per group) of *Anxa1*$^{fl/fl}$ and *Ggt-Cre*/*Anxa1*$^{fl/fl}$ mice (two-tailed Student's *t*-test). ANXA1 protein was almost absent in the knockout group, quantified residual

values reflect background noise and are defined as “undetectable”. (L) UACR, representative PAS photomicrographs and quantifications showing typical tubulointerstitial and glomerular structure in mice (n = 6, 8, 10, 9 respectively, two-way ANOVA followed by Tukey’s post hoc test). Scale bar:50 μm. (M) Quantifications of mean fluorescence intensity of UCP1 and GATA3 in the kidneys of 4 mice groups (n = 6, 8, 10, 9 respectively, two-way ANOVA followed by Tukey’s post hoc test). *$p < 0.05$, **$p < 0.01$, ***$p < 0.001$, ****$p < 0.0001$.

Figure S3

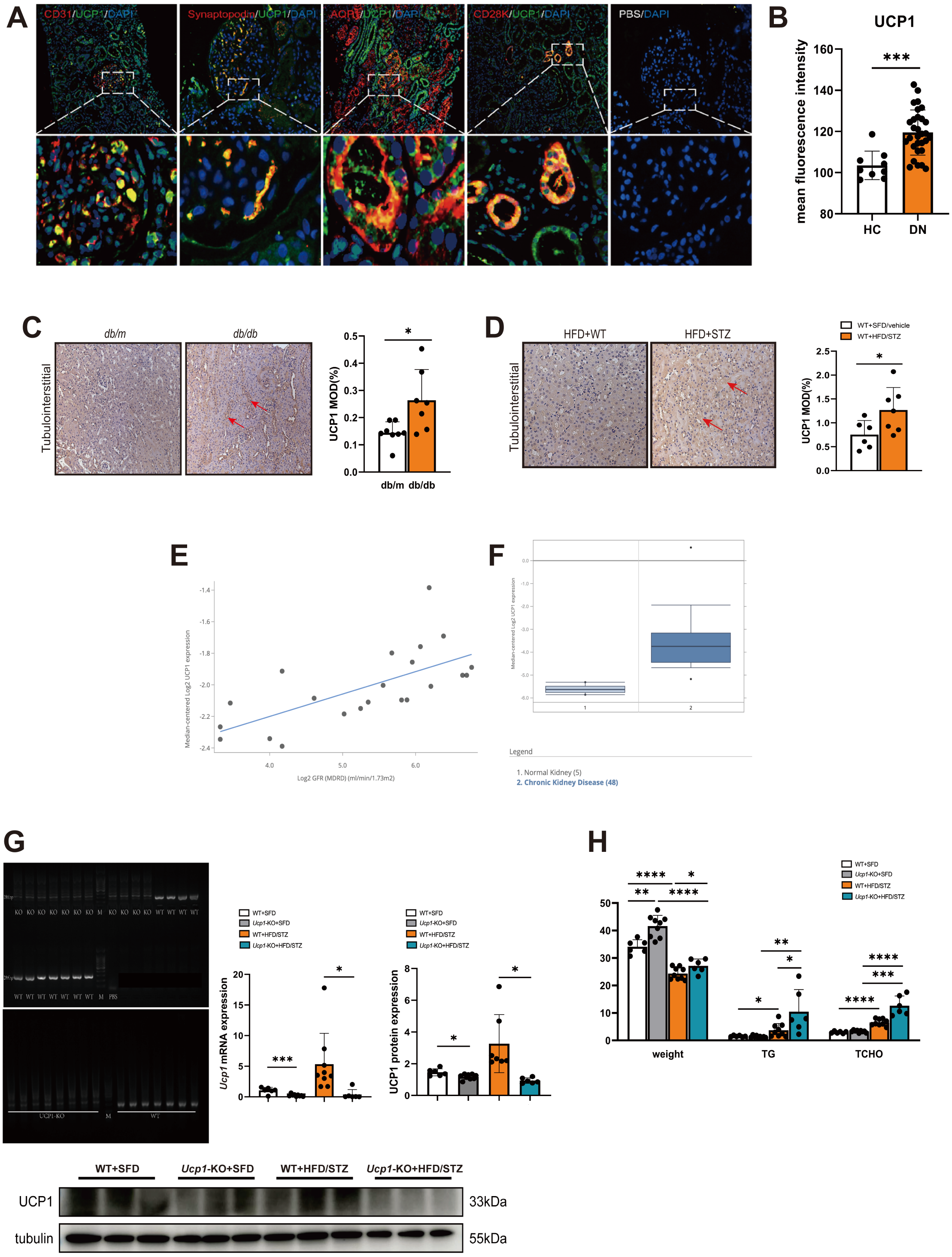

Figure S3. (A) Double immunofluorescence staining of UCP1 (green) and renal intrinsic cells (red); bar = 50 μm; magnified insets, original magnification, ×400. (B) Quantifications of mean fluorescence intensity of UCP1 in human renal cortical tissues

from DN (n = 30) and control (n = 9) in the Chinese cohort (two-tailed Student's *t*-test). (C) Representative IHC images and quantifications of UCP1 in the kidney from *db/db* (n = 7) and *db/m* mice (n = 8) (two-tailed Student's *t*-test). Scale bar: 50μm. (D) Representative IHC images and quantifications of UCP1 in the kidney from HFD/STZ mice (n = 7) and SFD/vehicle mice (n = 6) (two-tailed Student's *t*-test). Scale bar: 50μm. (E) Correlation between tubulointerstitial *UCP1* mRNA levels and GFR in DN patients from Woroniecka Diabetes TubInt dataset (n=22). (F) mRNA level of *UCP1* in kidney samples of patients with CKD (n=48) and normal kidney tissues (n=5) from Nakagawa CKD cohort. (G) Representative genotype identification and knockout efficiency validation in both mRNA and protein levels (n = 6, 9, 9, 6 respectively) of 4 mice groups. (H) Weight, TG and TCHO in 4 mice groups (n = 6, 9, 9, 6 respectively, two-way ANOVA followed by Tukey's post hoc test). $^{*}p < 0.05$, $^{**}p < 0.01$, $^{***}p < 0.001$, $^{****}p < 0.0001$.

Figure S4

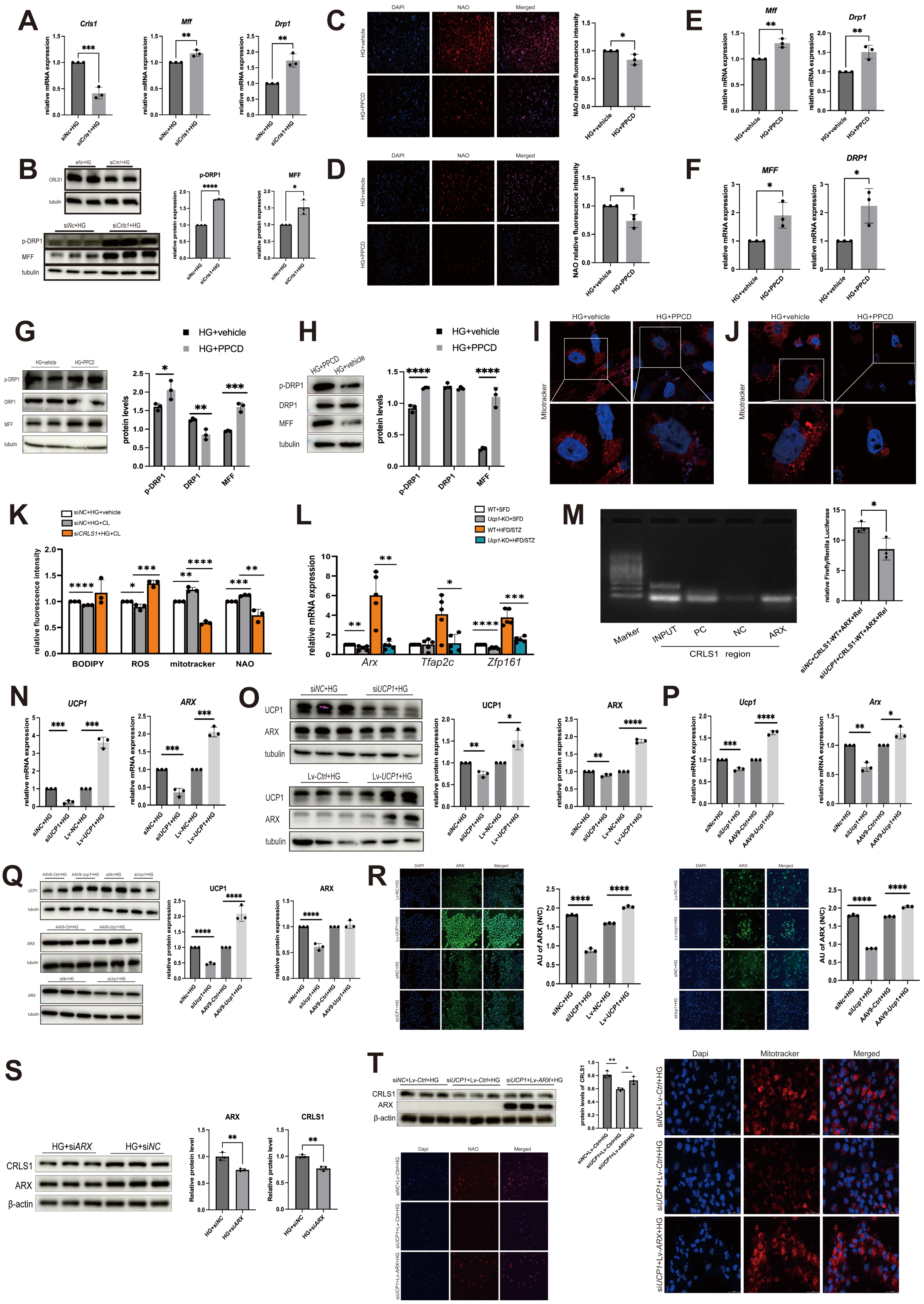

Figure S4. (A) Relative mRNA levels of *Crls1*, *Mff* and *Drp1* in primary murine PTECs upon *Crls1* silencing under HG condition (n = 3 per group, two-tailed Student's *t*-test).

(B) Representative blot images and quantifications of MFF and p-DRP1 in primary murine PTECs upon *Crls1* silencing under HG condition (n = 3 per group, two-tailed Student's *t*-test). (C) Representative images and quantifications of NAO staining in HK-2 cells upon PPCD treatment. (n = 3 per group, two-tailed Student's *t*-test). (D) Representative images and quantifications of NAO staining in primary murine PTECs upon PPCD treatment. (n = 3 per group, two-tailed Student's *t*-test). (E) Relative mRNA levels of *Mff* and *Drp1* in primary murine PTECs upon PPCD treatment (n = 3 per group, two-tailed Student's *t*-test). (F) Relative mRNA levels of *MFF* and *DRP1* in HK-2 cells upon PPCD treatment (n = 3 per group, two-tailed Student's *t*-test). (G) Representative blot images and quantifications of p-DRP1, DRP1 and MFF in primary murine PTECs upon PPCD treatment (n = 3 per group, two-tailed Student's *t*-test). (H) Representative blot images and quantifications of p-DRP1, DRP1 and MFF in HK-2 cells upon PPCD treatment (n = 3 per group, two-tailed Student's *t*-test). (I) Representative images of Mitotracker staining in primary murine PTECs upon PPCD treatment. (J) Representative images of Mitotracker staining in HK-2 cells upon PPCD treatment. (K) Representative images and quantifications of BODIPY, ROS, mitotracker and NAO probes by flow cytometry among 3 groups (n = 3 per group, one-way ANOVA followed by Tukey's post hoc test). (L) Relative mRNA level of *ARX, TFAP2C* and *ZFP161* in the kidneys of 4 mice groups (n = 5 per group, two-way ANOVA followed by Tukey's post hoc test). (M) ChIP assay using an anti-ARX antibody with primers for the putative region of the *CRLS1* promoter; dual-luciferase reporter assays for the CRLS1 promoter under *UCP1* deficiency. (N) Relative mRNA

levels of *UCP1* and *ARX* in HK-2 cells upon *UCP1* silencing and overexpressing under HG condition (n = 3 per group, two-tailed Student's *t*-test). (O) Representative blot images and quantifications of UCP1 and ARX in HK-2 cells upon *UCP1* silencing and overexpressing under HG condition (n = 3 per group, two-tailed Student's *t*-test). (P) Relative mRNA levels of *Ucp1* and *Arx* in primary murine PTECs upon *Ucp1* silencing and overexpressing under HG condition (n = 3 per group, two-tailed Student's *t*-test). (Q) Representative blot images and quantifications of UCP1 and ARX in primary murine PTECs upon *Ucp1* silencing and overexpressing under HG condition (n = 3 per group, two-tailed Student's *t*-test). (R) Representative immunofluorescence images show ARX (green) and nuclei (DAPI, blue) in four groups in HK-2 cells and primary murine PTECs. Nuclear translocation was quantified by calculating the Nuclear/Cellular fluorescence intensity ratio in individual cells (from 3 independent biological replicates). (S) Representative blot images and quantifications of CRLS1 and ARX in HK-2 cells upon *ARX* silencing under HG condition (n = 3 per group, two-tailed Student's *t*-test). (T) Representative blot images and quantifications of CRLS1 and ARX, together with cardiolipin staining and mitochondrial morphology in HK-2 cells among 3 groups under HG condition (n = 3 per group, one-way ANOVA followed by Tukey's post hoc test). $^{*}p < 0.05$, $^{**}p < 0.01$, $^{***}p < 0.001$, $^{****}p < 0.0001$.

Figure S5

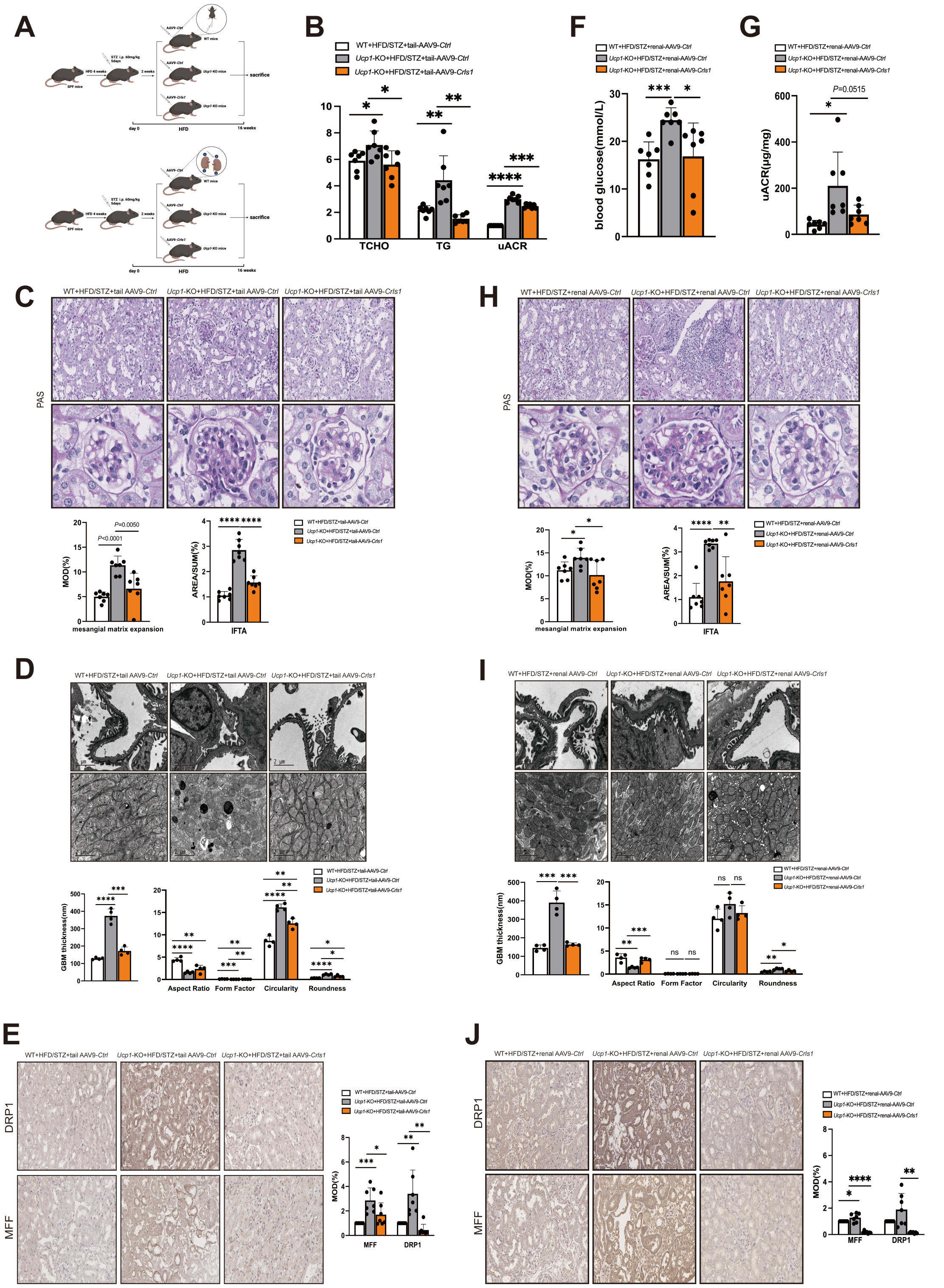

Figure S5. Renal overexpression of CRLS1 rescued kidney injury in HFD/STZ-induced diabetic *Ucp1*$^{-/-}$ mice. (A) A schematic diagram showing the 2 types of procedures of renal cortical AAV delivery in HFD/STZ-induced diabetic *Ucp1*$^{-/-}$ mice.

(B) TCHO, TG and uACR in mice (n = 7 per group, one-way ANOVA followed by Tukey's post hoc test). (C) Representative PAS photomicrographs and quantifications showing typical tubulointerstitial and glomerular structure in mice (n = 7 per group, one-way ANOVA followed by Tukey's post hoc test). Scale bar:50 μm. (D) Representative images and quantifications of TEM analyses in mice (n = 4, 4, 4 respectively, one-way ANOVA followed by Tukey's post hoc test). Scale bar: 2 μm. (E) Representative IHC images and quantifications of MFF and DRP1 in the kidneys of 3 mice groups (n = 7 per group, one-way ANOVA followed by Tukey's post hoc test). Scale bar: 50μm. (F) The level of blood glucose in 3 mice groups (n = 7 per group, one-way ANOVA followed by Tukey's post hoc test). (G) UACR in mice (n = 7 per group, one-way ANOVA followed by Tukey's post hoc test). (H) Representative PAS photomicrographs and quantifications showing typical tubulointerstitial and glomerular structure in mice (n = 7 per group, one-way ANOVA followed by Tukey's post hoc test). Scale bar:50 μm. (I) Representative images and quantifications of TEM analyses in mice (n = 4, 4, 4 respectively, one-way ANOVA followed by Tukey's post hoc test). Scale bar: 2 μm. (J) Representative IHC images and quantifications of MFF and DRP1 in the kidneys of 3 mice groups (n = 7 per group, one-way ANOVA followed by Tukey's post hoc test). Scale bar: 50μm. *$p < 0.05$, **$p < 0.01$, ***$p < 0.001$, ****$p < 0.0001$.

Figure S6

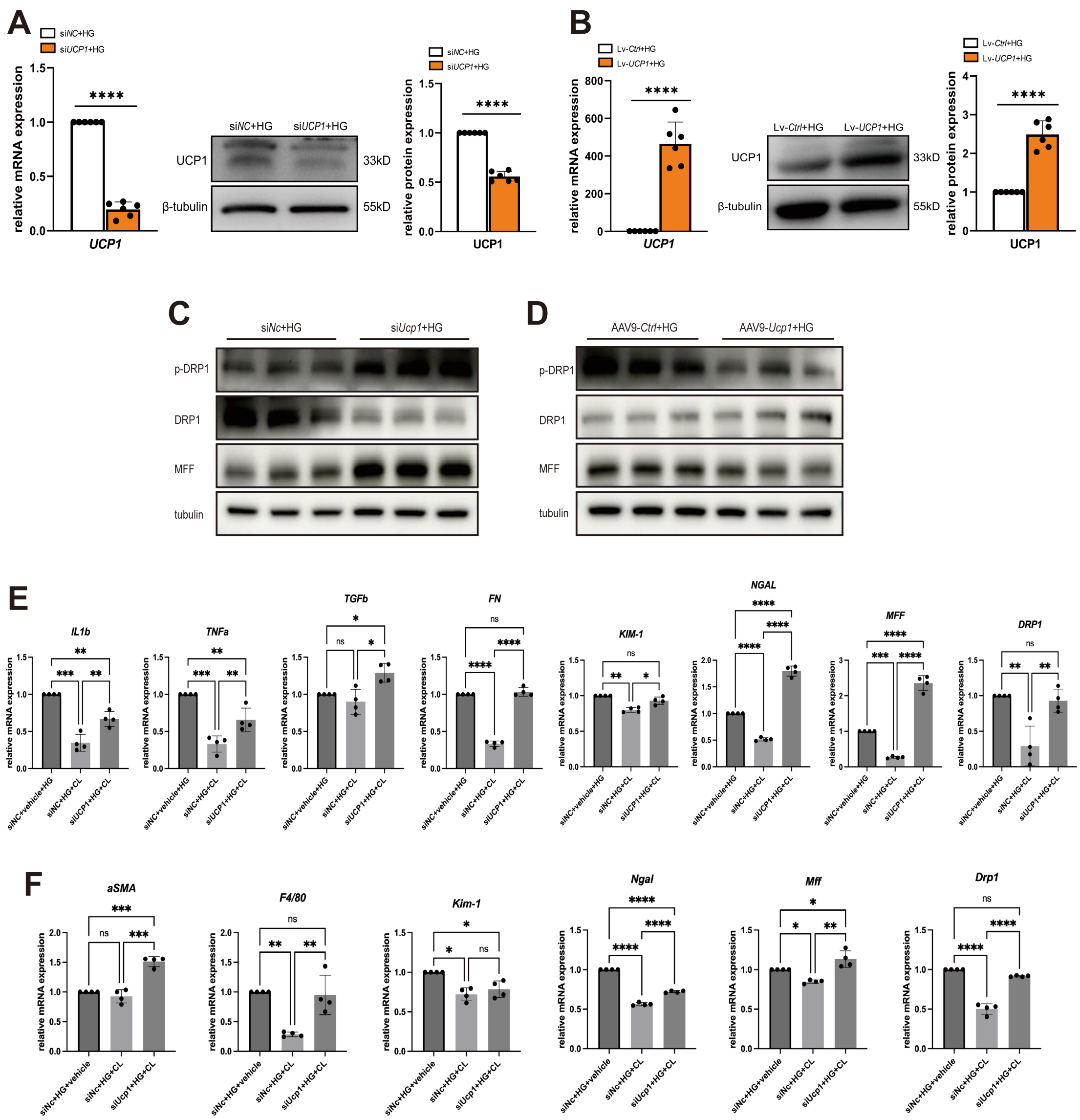

Figure S6. (A) Determination of *UCP1* knockdown efficiency by real-time quantitative PCR and western blot (n = 6 per group, two-tailed Student's *t*-test). (B) Determination of *UCP1* overexpression efficiency by real-time quantitative PCR and western blot (n = 6 per group, two-tailed Student's *t*-test). (C-D) Representative western blot (n = 3 blots in total) of p-DRP1, DRP1 and MFF upon *Ucp1* silencing and overexpressing under HG condition in primary murine PTECs (two-tailed Student's *t*-test). (E) Relative mRNA levels of *IL-1β*, *TNFα*, *TGFβ* and *FN*, *KIM-1*, *NGAL*, *MFF* and *DRP1* among

three groups in HK-2 cells (n = 3 per group, two-way ANOVA followed by Tukey's post hoc test). (F) Relative mRNA levels of *αSMA, F4/80, Kim-1*, *Ngal*, *Mff* and *Drp1* among three groups in primary murine PTECs (n = 3 per group, two-way ANOVA followed by Tukey's post hoc test). *p < 0.05, **p < 0.01, ***p < 0.001, ****p < 0.0001.
