# Supplementary material for: ANXA1 improves mitochondrial homeostasis through uncoupling protein 1 in diabetic nephropathy
Source: J Adv Res. 2025 Nov 22;86:689–708. doi: 10.1016/j.jare.2025.11.039 (PMC13453871; doi:10.1016/j.jare.2025.11.039)
Supplement: Supplementary file 2 [file mmc3.docx]

**Human Renal Biopsy Samples**

Thirty diabetes patients with biopsy-proven DN were enrolled, patients with other known kidney diseases were excluded. Normal control samples were obtained from the healthy kidney poles of individuals undergoing tumor nephrectomies without diabetes or kidney disease.

**Animal Studies**

Animals were grouped 3-5 per cage in a randomized manner and housed under standard temperature (24℃) and laboratory conditions (12h on/off; light on at 7:00 a.m.) with access to water and high-fat diet (HFD) or control standard-fat diet (SFD). Cages with standard corncob bedding were changed twice to three times a week. All items used by the mice were autoclaved, all of our experimental animals and operations were kept under barrier conditions. For all of our animal studies, littermate control mice were used. Considering that female mice are resistant to type 2 diabetes, only male mice were selected. For each experiment, the age and number of mice are showed in the figure legends. The designed genotype did not cause visible changes in initial weight, health or immune status.

**Generation of Proximal Tubular Epithelial Cells-Specific *Anxa1* Knockout Mice**

*Anxa1^fl/fl^* mice were hybridized with mice expressing Cre recombinase (Cre) under the control of the proximal tubular epithelial cell (PTEC) promoter to generate PTEC-specific *Anxa1* knockout mice (*PTEC-Cre Anxa1^fl/fl^* mice; *Cre^+^/Anxa1^fl/fl^* mice). Littermate homozygous *Anxa1^fl/fl^* mice without Cre expression were used as controls (*Cre^-^/Anxa1^fl/fl^* mice; *Anxa1^fl/fl^* mice). All the animals were in a C57BL/6J background. Mouse genotyping was performed using genomic DNA isolated from mouse toes by PCR at 2 weeks age with the following primers: flox genotyping primers: flox-forward: 5’-ATCAGCTCTTCTCCTCAGGTCC-3’; flox-reverse: 5’-TGGTGCAGCATAAAATGTGTGGTG-3’. The 195-bp and 162-bp fragments were produced for the mutant and wild type respectively. Wild type yields only a 162-bp band; homozygous (*Anxa1^fl/fl^*) yields only 195-bp band; heterozygous (*Anxa1^fl/+^*) yields both bands. Cre genotyping primers: Cre-forward: 5’-CATCACATCAGGCACCCCAGAA-3’; Cre-reverse: 5’-GAACATCTTCAGGTTCTGCGGGA-3’. Cre positive (*Cre^+^*) yields a 420-bp band, but Cre negative (*Cre^-^*) has no band. After genotyping, primary mice PTECs from *Cre^+^/Anxa1^fl/fl^* and *Anxa1^fl/fl^* mice were extracted and cultured according to protocols as described below, cell proteins were collected and the knockout efficiency of ANXA1 protein was verified by western blot.

**HFD/STZ-Induced DN in Mice**

Eight-week-old male mice were fed with a HFD diet (60% fat, HfkBio, China) for 1 month followed by an intraperitoneal injection of 60 mg/kg STZ (Sigma-Aldrich) dissolved in 50mM sodium citrate buffer (PH 4.5) for continuous 5days after starving for 6h to induce partial insulin deficiency. Mice were maintained on a HFD feeding for total 20weeks. Radom blood glucose was measured 14 days after STZ injection, and mice with random blood glucose > 16.7 mmol/L were considered successfully. Mice fed with a SFD diet (10% fat) and injected vehicle were used as non-diabetic controls. Blood glucose levels and body weight were monitored weekly, blood and 24h urine samples were collected for biochemical analysis every 2 to 4 weeks. At the end of the study, mice were euthanized and the kidney samples were harvested for histopathological analysis. The cortical parts of kidneys were dissected and collected for RNA or protein extraction for relevant analysis.

**Renal Cortical Adeno-Associated Virus Delivery in Mice**

The adeno-associated virus mouse *UCP1* expression plasmid was known as pGV388 contained target gene in the CMV bGlobin-MCS-EGFP-3FLAG-WPRE-Hgh-polyA with cloning site BamHI/BamHI (information:<http://www.genechem.com.cn/service/index.php?ac=gene&at=vector_search&keyword=GV388>); *CRLS1* expression plasmid (GV736 Ksp-cadherin-p-MCS-3flag-T2A-EGFP was designed by GeneChem Chemical Technology Co., Ltd (Shanghai, China). Each of them was delivered into the mouse kidney cortex by intraparenchymal injection 15 days after STZ injection. Before the surgery, mice were temporarily anesthetized, and then were placed in a prone position after sterilizing and skin preparation. The skin and subcutaneous tissue were cut along the dorsal midline, and muscles were cut at the costovertebral angle to expose the unilateral kidney. Then, 4µL of *UCP1*/*CRLS1* or control AAV (6.45E+12; 4.91E+13 v.g.mL^-1^, respectively) was injected in a site by a 23G needle. Two to four sites were selected from each kidney to slowly inject the AAV into the renal cortex. The kidney was returned after injection and the muscles were sutured completely. The same went for the other kidney. The skin is glued with medical glue after both kidneys were operated. Finally, the mice were placed on a thermal blanket machine until they woke up.

**Cell Culture and Treatment**

HK-2, human immortalized proximal tubular epithelial cells (PTECs), were purchased from American Type Culture Collection. Cells were cultured in DMEM/nutrient mixture F-12 and enriched with 10% fetal bovine serum and 1% penicillin-streptomycin under 5% CO_2_ at 37℃. At 80% confluence, the cells were stimulated with high glucose at final concentration of 30 mmol/L for 72h. The control group was set as the isotonic solvent using mannitol. To upregulated *UCP1*/UCP1, cells were treated with CL316243 (0.3µmol/L; [MedChemExpress](mailto:MCE%3Csales@MedChemExpress.cn%3E)), human recombinant ANXA1 (hrANXA1, 10nmol/L; R&D systems) or Ac2-26 (15µmol/L; R&D systems). For the protease inhibition experiments, cells were treated with Cycloheximide (CHX, 100µmol/L, [MedChemExpress](mailto:MCE%3Csales@MedChemExpress.cn%3E)), with or without MG-132 (50µmol/L, [MedChemExpress](mailto:MCE%3Csales@MedChemExpress.cn%3E)) for 0, 1, 2, 4 and 6 hours. For cardiolipin biosynthesis inhibition, cells were treated with 3,5-dimethylpyrazole-pyridine-chlorodinitrobenzene conjugate (PPCD, 10 µmol/L, Sigma-Aldrich) for 48 hours. HK-2 cells and primary mouse PTECs were cultured under standard HG conditions prior to PPCD exposure.

TCMK-1, mouse kidney epithelial cell line, was purchased from Fuheng Biotechnology (China). Cells were cultured in MEM and enriched with 10% fetal bovine serum and 1% penicillin-streptomycin under 5% CO_2_ at 37℃.

Primary mouse proximal tubular epithelial cells (MPTECs) were seeded in 6 or 12-well plates as needed and cultured in Epithelial Cell medium (EpiCM-A; ScienCell, United States) supplemented with 2% FBS (ScienCell), 1% penicillin-streptomycin (ScienCell) and 1% EpiCGS-a (ScienCell) in 37° C in an incubator containing 5% CO_2_ and 95% humidified air. When reaching approximately 70% confluence, the medium was replaced for the first time, when it reached to almost 95% confluence, the MPETCs can then be collected for further experiments as propriate.

**Histological Analysis of Renal Samples**

Renal biopsy kidney tissues and mouse kidneys were transferred to 4% paraformaldehyde (PFA) contained in a fresh tube and fixed overnight. Tissues were embedded in paraffin and cross-sectioned (4µm) for histology examination. Periodic-acid-Schiff (PAS) was used to stain the kidney sections according to manufacturer’s instructions by using staining kits. For patients, the degree of glomerulosclerosis is defined as the percentage of the total numbers of glomerulosclerosis. Tubulointerstitial inflammation was scored semi-quantitatively, based on the percentage of the tubulointerstitial compartment affected (0: none, 1: < 25%, 2: 25-50%, 3: > 50%). For mice kidneys, mesangial matrix expansion area was assessed from the digital photographs of glomeruli and was defined as the PAS-positive and nuclei-free area and calculated as a ratio to a total glomerular area. The tubulointerstitial injury index was determined by assessing the severity and amount and tubule dilation, atrophy and loss of tubular cells, and was scored as follows: 0: no injury, 1: < 25%, 2: 25-50%, 3: 50-75%, 4: > 75% of the proportion with tubulointerstitial injury. Histological analysis was performed in a blinded fashion. At least six glomeruli per human or ten glomeruli per mice in each section were photographed at 200X magnification. Quantitation analysis was done on Image-Pro Plus.

**Immunohistochemistry and Immunofluorescence**

Paraffin-embedded kidney sections (4µm) were stained with different primary antibodies. The sections were incubated with peroxidase-conjugated secondary antibodies after incubating with primary antibody at 4℃ overnight according to the instructions by using their kits (ZSGB-BIO, Beijing). Sections were digitized with a high-resolution camera.

Immunofluorescence staining was performed using primary antibodies we were interested. Sections were incubated with different primary antibodies mentioned above, and subsequently incubated with secondary Alexa 488 or 555 conjugated antibody (Invitrogen). Nuclei were stained with DAPI (ZSGB-BIO, Beijing). Antibodies used in this part are showed in Key Resources Table (Table S1).

**Transmission Electron Microscopy**

Kidney tissues were fixed in 3% glutaraldehyde at 4℃, followed by dehydration in graded ethanol and washed with 100% acetone in series, and embedded in Epon 812 overnight in 37℃ finally. The ultra-thin sections were cut and stained with uranyl acetate and lead citrate. TEM images were photographed by a transmission electron microscope (Tecnai G^2^ 20 TWIN; FEI, USA). The glomerular basement membrane (GBM) thickness was measured by Image-Pro Plus software. All glomeruli were selected from each mouse kidney sample and ten electron micrographs were taken in each glomerulus.

**Real-time Quantitative Polymerase Chain Reaction Analysis**

Total RNA was extracted from cultured cells or mouse kidney cortex using kits, cDNA was synthesized by using the PrimeScriptTM RT reagent kit (Takara, Japan) according to the manufacturer’s instruction. Real-time qPCR was performed using SYBR Green reagent (A25742; Applied Biosystems). The mRNA levels were analyzed by a QuantStudio system. Levels of the 18S ribosomal RNA or β-actin were used as an internal control for normalization. The results were shown as fold changes compared to the control group by the ΔΔCT method.

**Flow Cytometry**

Intracellular reactive oxygen species (ROS) levels were measured by commercial kits (Beyotime) according to the manufacturer’s instructions. BODIPY™ 581/591 C11 (D3861, Invitrogen) was used to determine the lipid peroxidation levels in cells by following the protocol. Acridine Orange 10-Nonyl Bromide (NAO) is a fluorescent probe for cardiolipin (HY-D0993, [MedChemExpress](mailto:MCE%3Csales@MedChemExpress.cn%3E)), which was used to examined the content of cardiolipin in cells for different conditions. MitoTracker (Beyotime) can be carried out to detect bioactive mitochondria in cells. The above indicators were not only observed and analyzed in coverslips planted with cells by high resolution microscope, but also detected by flow cytometry according to the manufacturer’s instructions.

**Western Blot**

Mice kidney tissues as well as cultured cells were resuspended in RIPA buffer (150 mM NaCl, 0.5% sodium deoxycholate, 0.1% SDS, 1% NP40, 1 mM EDTA and 50 mM Tris pH 8.0). Equal concentration proteins were separated by sodium dodecyl sulfate polyacrylamide gel electrophoresis and followed by being transferring to PVDF membrane and blocking with 5% milk for 1 to 2 hours in room temperature. Then, the membranes were incubated with primary antibodies summarized in Key Resources Table at 4℃ overnight and subsequently incubated with secondary antibodies at room temperature for an hour. Proteins were visualized on autoradiographic film using an ECL Plus Western blot detection system (GE Healthcare).

**Isolation of Mice Primary Renal Proximal Tubular Cells (MPETCs)**

In brief, kidneys were immediately harvested and washed in sterile ice-cold PBS. Each kidney was decapsulated, bisected, and the inner medullary portion was excised. The remaining cortical and outer medullary regions were pulverized, and mixed in a solution of 30 mL of PBS containing 1 mg/mL collagenase (type Ⅱ; Invitrogen, Carlsbad, CA, USA) for every 1 g of minced kidney. The solution was incubated for 10 min at 37°C in a shaker machine in a gentle manner, followed by collecting the supernatant after 10s silent placement. Supernatant was terminated by equal volume dilution EpiCM-A medium and placed on ice. The remaining tissue precipitates were added with new PBS-collagenase mixture, repeat the steps above 3-4 times. The collected supernatant was centrifuged at 4°C, 500g for 5 minutes, then the supernatant was abandoned and the deposits were resuspended with 25ml ice-cold PBS. The centrifugation was repeated twice. The final supernatant was abandoned and the primary MPTECs were resuspended by complete medium for further use.

**Biochemical Analysis of Serum/Urine Samples**

Body weight, kidney weight and fasting blood glucose were measured. Serum total triglyceride, cholesterol concentrations and urine creatinine levels were analyzed by commercial kits. Urine albumin concentrations were measured by Bethyl Laboratories kit. The level of urine albumin was normalized to urine creatinine (uACR).

**TUNEL Apoptosis Assay**

Cell death was determined using TUNEL Apoptosis Assay Kit (Beyotime) according to the manufacturer’s instructions. Ten random fields were selected to access the percentage of apoptotic cells by ImageJ.

**Mitochondrial Visualization**

Mitochondria were visualized using MitoTracker (Beyotime) according to the manufacturer’s instructions. Mitochondria network morphology was skeletonized and followed by measurement of individuals, networks, mitochondrial footprint and mean branches per network using the Mitochondrial Network Analysis toolset on Fiji (ImageJ). Fifteen random fields were examined per sample.

**Oxygen Consumption Rate Assay**

Mitochondrial function was explored by a Seahorse flux analyzer (Agilent Technologies) according to the manufacturer’s instructions. Briefly, 2 × 10^5^ cells/ well were seeded into XF24 cell culture microplates and cultured in growth medium (Agilent Technologies). Before the assay, the cells were rinsed with assay medium. For FAO assay, HG or mannitol was introduced into appropriate wells. Oligomycin (2 mmol/L), carbonyl cyanide p-trifluoromethoxyphenylhydrazone (1 mmol/L), and rotenone/antimycin A (0.5 mmol/L) were sequentially added into the culture, and oxygen consumption rate (OCR) was assessed.

**Oil Red O Staining**

The frozen sections of mouse kidney cortex were prepared and air-dried at room temperature. HK-2 cells were cultured on coverslips and fixed in 4% PFA. Oil Red O staining was performed by kit (Solarbio) according to the manufacturer’s instructions. In brief, sections or coverslips were dipped into 60% isopropanol three times and the stained with 0.3% Oil Red O dye solution also dissolved in 60% isopropanol for 10 min. The sections or coverslips were washed with 75% isopropanol for 1 min and counterstained with hematoxylin for 1 min, then were mounted with microscope cover glassed and slides respectively after washing. The deposition of lipid droplets was observed by microscope. Images were analyzed with Image-Pro Plus.

**Co-immunoprecipitation (Co-IP)**

Co-IP was performed using a Pierce Crosslink Magnetic Co-IP kit (#88804, ThermoFisher Scientific). Cells were lysed and the protein concentrations were carried out by BCA protein assay. As recommended, 500 µg of protein in 500 µL supernatant was incubated with 5µg anti-UCP1, anti-ANXA1, anti-TRX or anti-IgG antibodies coated on beads in a rotator at 4℃ overnight. The beads were washed to remove non-bound material and eluted in a low-PH elution buffer that could dissociate bound antigen from the antibody-crosslinked beads. The precipitate was separated by SDS-PAGE and detected by western blot.

**Chromatin Immunoprecipitation (CHIP) Assay**

TCMK-1 were incubated in 1% paraformaldehyde to fix cells and crosslink chromatin. Then supernatants were sonicated on ice at 30% amplitude for a total of 10 times and 10s each time on an ultrasonic machine to obtain sheared chromatin, followed by analyzing for the enrichment of the GATA3 protein on the proximal region (−140 ~ +1) and the distal enhancer region (−2500 to −2400) of the *UCP1* promoter region. Immunoprecipitated DNA fragments were analyzed by Real-Time PCR using the following primers: *Ucp1* proximal region (5’-CCCACTAGCAGCTCTTTGGA-3’ and 5’-CTGTGGAGCAGCTCAAAGGT-3’) and *Ucp1* distal enhancer region (5’-CTCCTCTACAGCGTCACAGAGG-3’ and 5’-AGTCTGAGGAAAGGGTTGA-3’). Immunoprecipitation was performed using anti-GATA3 antibody (for GATA3 IP), anti-Histone H3 antibody (positive control, PC), or rabbit IgG (negative control, NC), while a fraction of total chromatin was saved as INPUT.

**Luciferase Assay**

The mouse *Ucp1* proximal promoter region (−98 ~+81 bp) was cloned into the pGV238 firefly luciferase reporter vector (information: <http://www.genechem.com.cn/service/index.php?ac=gene&at=vector_search&keyword=GV238>) to generate the *Ucp1*-luc construct. The mouse *Gata3* coding sequence was cloned into the pGV712 expression vector (information:<http://www.genechem.com.cn/service/index.php?ac=gene&at=vector_search&keyword=GV712>**)** to generate the *Gata3*-exp construct. Corresponding empty vectors (con520 for pGV238 and con080 for pGV712) were used as negative controls. For the dual-luciferase assay, TCMK-1 cells were co-transfected with the indicated plasmids using a commercial transfection reagent (GenChem Co., Ltd Shanghai, China). A Renilla luciferase plasmid was co-transfected in all groups as an internal control (GenChem Co., Ltd Shanghai, China). Cell lysates from transfected cells were extracted using lysis buffer and luciferase activity was measured using a luciferase reporter assay kit (PROMEGA, E1910). Relative luciferase activity was expressed as the ratio of firefly to Renilla luciferase activity.

**Lentivirus-Mediated Gene Expression and Interference**

The lentivirus human *ANXA1* expression plasmid was known as pGV358 contained target gene in the Ubi-MCS-3FLAG-SV40-EGFP-IRES-puromycin vector (information:<http://www.genechem.com.cn/service/index.php?ac=gene&at=vector_search&keyword=GV358>); the lentivirus human *UCP1* expression plasmid was known as pGV341 contained target gene in the Ubi-MCS-3FLAG-SV40-puromycin vector (information:<http://www.genechem.com.cn/service/index.php?ac=gene&at=vector_search&keyword=GV341>); the lentivirus human *GATA3* expression plasmid was known as pCV572 contained target gene in Ubi-MCS-SV40-Cherry-IRES-neomycin vector (information:<http://www.genechem.com.cn/index/supports/tool_search.html?keywords=CV572>); The lentivirus sgRNA human *UCP1* sequence (TCGGACGTACACCCGACCCT) was cloned to get a pGV392 plasmid contained gene in U6-sgRNA-EF1a-Cas9-FLAG-P2A-puromycin vector (information: [http://www.genechem.com.cn/service/index.php?ac=gene&at=vector_search&keyword=GV392](http://www.genechem.com.cn/service/index.php?ac=gene&at=vector_search&keyword=GV39241)). pGV358-*ANXA1*, pGV341-*UCP1*, pCV572-*GATA3*, Pgv392-*UCP1*-RNAi and their negative controls were generated and packaged from GenChem Co., Ltd (Shanghai, China). Stably *ANXA1*/*UCP1*/*GATA3*-overexpressed HK-2 cells and *UCP1* -silenced HK2 cells were established using lentivirus mentioned above according to the recommended infection methods. Cells were screened and kept in the medium containing 2µg/mL puromycin (Sigma-Aldrich) or 500µg/mL geneticin (G-418).

**Short Interfering RNA (siRNA)-mediated Knockdown and DNA Transfections**

One day before transfection, cells were plated and grown to ~50% confluence. When transfected, cells were cultured in medium without antibiotics and FBS. HK-2 cells were delivered with siRNAs (50nmol/L) (Ribo Bio Co., Ltd.) targeting human *ANXA1*/*GATA3*/*UCP1*/*CRLS1* genes by the Lipofectamine 3000 reagent (Invitrogen, Carlsbad, CA) following the manufacturer’s protocol, and the corresponding negative control siRNA was transfected as the control group.

**Cardiolipin Content Analysis**

Briefly, tissues were homogenized in 750 µL of chloroform: methanol: MilliQ H_2_O (3:6:1) (v/v/v). The homogenate was then incubated at 1500 rpm for 1h at 4℃. At the end of the incubation, 350 µL of deionized water and 250 µL of chloroform were added to induce phase separation. The samples were then centrifuged and the lower organic phase containing lipids was extracted into a clean tube. Lipid extraction was repeated once by adding 450 µL of chloroform to the remaining aqueous phase, and the lipid extracts were pooled into a single tube and dried in the SpeedVac under OH mode. Samples were stored at -80℃ until further analysis. Lipidomic analyses were conducted at LipidALL Technologies using a ExionLC-AD coupled with Sciex QTRAP 6500 PLUS as reported previously^[17]^. Separation of individual lipid classes of polar lipids by normal phase (NP)-HPLC was carried out using a TUP-HB silica column (i.d. 150x2.1 mm, 3 µm) with the following conditions: mobile phase A (chloroform: methanol: ammonium hydroxide, 89.5:10:0.5) and mobile phase B (chloroform: methanol: ammonium hydroxide: water, 55:39:0.5:5.5). MRM transitions were set up for comparative analysis of various polar lipids. Individual lipid species were quantified by referencing to spiked internal standards. d_9_-PC32:0(16:0/16:0), d_9_-PC36:1p(18:0p/18:1), d_7_-PE33:1(15:0/18:1), d_9_-PE36:1p(18:0p/18:1), d_31_-PS(d31-16:0/18:1), d_7_-PA33:1(15:0/18:1), d_7_-PG33:1(15:0/18:1), d_7_-PI33:1(15:0/18:1), C17-SL, d_5_-CL72:8(18:2)4, d_7_-LPE18:1, C17-LPI, C17-LPA, C17-LPS, were obtained from Avanti Polar Lipids. GM3-d18:1/18:0-d_3_ was purchased from Matreya LLC. Free fatty acids were quantitated using d_31_-16:0 (Sigma-Aldrich) and d_8_-20:4 (Cayman Chemicals).

**Non-Targeted Metabolomics**

Proteins in the samples were precipitated by vortexing for 3 min at 4-8 °C, the supernatant (300µL) was recovered by centrifugation at 20,000 × g at 4 °C for 30 min. Then the supernatant was vacuum concentrated at room temperature for 3h. After that reconstituted with acetonitrile/water (3/1) solution. After 3 min vortexing, the mixture was centrifuged at 20,000 × g at 4 °C for 15min.Then, the supernatants were used for LC-MS analysis. The LC−MS/MS analyses were conducted using an Nexera XR LC-20ADXR system (Shimadzu Europa, Duisburg, Germany) and an AB SCIEX TripleTOF 5600+ system (AB SCIEX, Foster City, USA). A Waters ACQUITY HSS T3 column (2.1 ×100 mm, 1.8 μm; Waters, Milford, MA, USA) was used for the separation. Reagent grades for metabolomic analysis were purchased from Fisher Chemicals (Marshalltown, USA). The injection volume was 3 μL for positive ESI mode and 5 μL for negative ESI mode. For both ion modes, the column temperature was maintained at 35 °C. A gradient program was used at a flow rate of 250 μL/min; mobile phase A was water containing 0.1% formic acid and mobile phase B was pure acetonitrile. The gradient program was applied as follows: 98% A/2% B for 0-0.1 min; increased to 40% A/60% B from 0.1 to 5 min; maintained at 40% A/60% B from 5 to 10 min; increased to 0% A/100% B from 10 to 17 min; maintained at 0% A/100% B from 17 to 20 min; decreased to 98% A/2% B from 20 to 20.1 min; The stop time was 0.1 min. For the acquisition of the MS/MS data, the MS system was operated using an AB SCIEX TripleTOF 5600 mass analyzer with information dependent acquisition (IDA) for ions in both positive and negative modes. For positive mode, the collision energy was set to 10 V, declustering potential was set to 80 V, and ion spray voltage was set to +5500 V with mass range from 100 to 1000 m/z. For negative mode, the collision energy was set to −10 V, declustering potential was set to−80 V, and ion spray voltage was set to −4500 V with a mass range from 100 to 1000 m/z. The other source parameters were as follows: the ion source gas 1 was set at 50 psi, the ion source gas 2 was set at 55 psi, the curtain gas was set at 35 psi, and the drying temperature was set at 450°C. The instrument was mass calibrated using Calibration Solution for AB SCIEX TripleTOF 5600 (part NO. 4460131) for ESI positive mode and Calibration Solution for AB SCIEX TripleTOF 5600 (part NO. 4460134) for ESI negative mode.
