# Supplementary material for: ANXA1 improves mitochondrial homeostasis through uncoupling protein 1 in diabetic nephropathy
Source: J Adv Res. 2025 Nov 22;86:689–708. doi: 10.1016/j.jare.2025.11.039 (PMC13453871; doi:10.1016/j.jare.2025.11.039)
Supplement: Supplementary file 3 [file mmc5.docx]

**KEY RESOURCES TABLE**

| **REAGENT or RESOURCE** | **SOURCE** | | **IDENTIFIER** |
| --- | --- | --- | --- |
| **Antibodies** | | | |
| Rabbit monoclonal anti-ADRP/Perilipin 2 | | Abcam | Cat#ab108323 |
| Rabbit monoclonal anti-DRP1 | | Abcam | Cat#ab184247 |
| Rabbit monoclonal anti-Mitofusin 2 | | Abcam | Cat#ab124773 |
| Rabbit monoclonal anti-OPA1 | | Abcam | Cat#ab157457 |
| Rabbit monoclonal anti-MFF | | Abcam | Cat#ab129075 |
| Rabbit monoclonal anti-GATA3 - ChIP Grade | | Abcam | Cat#ab199428 |
| Rabbit monoclonal anti-UCP1 | | Abcam | Cat#ab209483 |
| Rabbit monoclonal anti-mtTFA | | Abcam | Cat#ab176558 |
| Rabbit monoclonal anti-Annexin A1/ANXA1 | | Abcam | Cat#ab214486 |
| Rabbit polyclonal anti-UCP1 | | Abcam | Cat#ab10983 |
| Rabbit polyclonal anti-UCP2 | | Abcam | Cat#ab97931 |
| Rabbit polyclonal anti-UCP3 | | Abcam | Cat#ab10985 |
| Rabbit polyclonal anti-UCP1 | | Abcam | Cat#ab155117 |
| Rabbit monoclonal β-Tubulin (9F3) | | Cell signaling technology | Cat#2128T |
| Mouse monoclonal Parkin (Prk8) | | Cell signaling technology | Cat#4211T |
| Rabbit monoclonal PGC-1α (3G6) | | Cell signaling technology | Cat#2178S |
| Rabbit monoclonal Phospho-DRP1 (Ser616) | | Cell signaling technology | Cat#4494S |
| Mouse monoclonal anti-Synaptopodin | | R&D Systems | Cat# MAB8977 |
| Mouse monoclonal anti- Mitofusin 1 | | Santa Cruz biotechnology | Cat#sc-166644 |
| Mouse monoclonal anti-PECAM-1 | | Santa Cruz Biotechnology | Cat#sc-53411 |
| Mouse monoclonal anti-integrin-α8 | | Santa Cruz Biotechnology | Cat#sc-365798 |
| Mouse monoclonal anti-AQP1 | | Santa Cruz Biotechnology | Cat#sc-25287 |
| Mouse monoclonal anti-CD28K | | Santa Cruz Biotechnology | Cat#sc-365360 |
| Rabbit polyclonal anti-MFF | | HUABIO | Cat#ER1902-93 |
| Rabbit monoclonal anti-hospho-GATA3 (S308) | | HUABIO | Cat#ET1609-17 |
| Rabbit polyclonal anti-CRLS1 | | Proteintech | Cat#14845-1-AP |
| Rabbit polyclonal anti-Thioredoxin | | Proteintech | Cat#14999-1-AP |
| HRP-conjugated Affinipure Goat Anti-Rabbit IgG(H+L) | | Proteintech | Cat#SA00001-2 |
| HRP-conjugated Affinipure Goat Anti-Mouse IgG | | Proteintech | Cat#SA00001-1 |
| Alexa Fluor 488-labeled donkey anti-rabbit IgG | | ImmunoResearch | Cat#711-545-152 |
| cyanin 3-labeled donkey anti-mouse IgG Jackson | | ImmunoResearch | Cat#715-165-150 |
| Normal rabbit IgG | | Cell signaling technology | Cat#3900S |
| **Chemicals, Peptides, and Recombinant Proteins** | | | |
| Streptozocin | | Sigma-Aldrich | Cat# S0130 |
| High-fat diet (60 kcal% from fat) | | Research Diets | Cat# D12492 |
| Penicillin/Streptomycin | | Gibco | Cat#15140-122 |
| Fetal bovine serum | | Gibco | Cat# 10099141 |
| Mannitol | | Sigma-Aldrich | Cat# M4125 |
| Oil Red O | | Solarbio | Cat# G1261 |
| D- (+) Glucose | | Sigma-Aldrich | Cat# G8270 |
| 100mM PMSF | | Beyotime Biotechnology | Cat# P0023A |
| Lipofectamine 3000 transfection reagent | | Invitrogen | Cat# 11668500 |
| human recombinant ANXA1 | | R&D | Cat# 3770-AN-050 |
| Primary Antibody Dilution Buffer | | Beyotime Biotechnology | Cat# P0023A |
| Marker Ⅰ DNA Ladder | | Solarbio | Cat# M1100-100T |
| Acridine Orange 10-Nonyl Bromide | | MedChemExpress | Cat#HY-D0993 |
| Puromycin Dihydrochloride | | Beyotime Biotechnology | Cat# ST551 |
| Stripping Buffer | | Qualityard | Cat# HX18672 |
| 2×Taq PCR Master mix | | TIANGEN | Cat# KT201-02 |
| Ac2-26 peptide | | R&D | Cat# 1845 |
| DNase I (RNase Free) | | Accurate Biology | Cat# AG12001 |
| 3M Vetbond Tissue Medical Glue | | 3M | Cat# 1469SB |
| WRW4 | | meilunbio | Cat# MB1872 |
| Gel-Red | | Beyotime Biotechnology | Cat# D0139 |
| Dulbecco's Modified Eagle Medium/Nutrient Mixture F-12 | | ThermoFisher | Cat#11330032 |
| MG-132 | | MedChemExpress | Cat# HY-13259 |
| Cycloheximide | | MedChemExpress | Cat# HY-12320 |
| 50×TAE Buffer | | Solarbio | Cat# T1060-500 |
| Oil Red O Staining Solution (cell only) | | Solarbio | Cat# G1262 |
| SDS-PAGE Protein Loading Buffer（5X） | | Beyotime Biotechnology | Cat# P0015L |
| MicroAmp™ Fast Optical 96-Well Reaction Plate with Barcode | | Thermo Scientific | Cat# 4346906 |
| 100bp DNA Ladder | | TransGen Biotechnology | Cat# BM301-01 |
| Hoechst 33342 (100X) | | Beyotime Biotechnology | Cat# C1028 |
| CL 316243 hydrate | | Sigma-Aldrich | Cat# C5976 |
| RIPA Lysis Solution | | Applygen | Cat# C1053-100 |
| POWRUP SYBR MASTER MIX | | Thermo Scientific | Cat# A25742 |
| Sodium Citrate Buffer | | Solarbio | Cat# Z9908 |
| Epithelial Cell Medium-animal | | ScienCell | Cat# 4131 |
| Applied Biosystems™ MicroAmp™ | | Thermo Scientific | Cat# 4360954 |
| XF DMEM medium | | Aglient | Cat# 103575-100 |
| XF 1.0 M glucose solution | | Aglient | Cat# 103577-100 |
| XF 100 mM pyruvate solution | | Aglient | Cat# 103578-100 |
| XF 200 mM glutamine solution | | Aglient | Cat# 103579-100 |
|  | |  | |
| **Critical Commercial Assays** | | | |
| Periodic Acid Schiff | | BASO | Cat# BA-4080A |
| Immobilon Western Chemiluminescent HRP substrate | | Millipore | Cat# WBKLS0100 |
| One Step Mouse Genotyping Kit | | Vazyme Biotech | Cat# PD101-01 |
| SteadyPure Rapid RNA Etraction Kit | | Accurate Biology | Cat# AG21023 |
| Pierce Classic Magnetic IP/Co-IP Kit ThermoFisher | |  | Cat# 88804 |
| BioSci® New Flash Protein AnyKD PAGE | | Dakewe Biotech | Cat# 8012011 |
| PrimeScript RT reagent Kit (Perfect Real Time) | | TaKaRa | Cat# RR037A |
| Luciferase Reporter Assay System | | Promega | Cat# E1910 |
| Mouse Albumin ELISA | | Bethyl | Cat# E99-134 |
| Total Cholesterol (TC) Assay Kit | | Nanjingjiancheng Biotech | Cat# A111-1-1 |
| Triglycerides (TG) Assay Kit | | Nanjingjiancheng Biotech | Cat# A110-1-1 |
| Creatinine (Cr) Assay Kit | | Nanjingjiancheng Biotech | Cat# C011-2-1 |
| TransDirect Animal Tissue PCR Kit | | Transgen Biotech | Cat# AD201-02 |
| IHC kit (anti-rabbit secondary antibody) | | ZSBIO | Cat# PV-9001 |
| 3,3′-diaminobenzidine DAB kit | | ZSBIO | Cat# ZLI-9018 |
| IHC kit (anti-mouse secondary antibody) | | ZSBIO | Cat# PV-9005 |
| High-Capacity cDNA Reverse Transcription Kit | | Thermo Scientific | Cat# 4368814 |
| RNAprep Pure Cell / Bacteria Kit | | TIANGEN | Cat# DP430 |
| Seahorse XFe24 FluxPak Kit | | Aglient | Cat# 102342-100 |
| Seahorse XF cell mitochondrial stress test kit | | Aglient | Cat# 103015-100 |
| **Experimental Models: Cell Lines** | | | |
| Human: HK-2 | | ATCC | Cat# CRL-219 |
| Mouse: TCMK-1 | | Fuheng Biotechnology | Cat# FH1286 |
|  | |  |  |
| **Experimental Models: Organisms/Strains** | | | |
| Mouse: C57BL/6J | | GemPharmatech (Jiangsu, China) | Cat# N00013 |
| Mouse:C57BLKS/JGpt-Leprem2Cd/Gpt (*db/db*) | | GemPharmatech (Jiangsu, China) | Cat# T002407 |
| Mouse: *Anxa1*-KO (C57BL/6J) | | Cyagen Biosciences Inc. (Jiangsu, China) | Contract No. KOTS150312CF1 |
| Mouse: *UCP1*-KO (C57BL/6J) | | GemPharmatech (Jiangsu, China) | Cat# T037633 |
| Mouse: ANXA1 flox (C57BL/6J) | | ViewSolid Biotech (Beijing, China) | Contract No. Fw2015121701BM |
| Mouse: Tg (Ggt-cre) M3Egn (C57BL/6N) | | Cyagen Biosciences  Inc. (Jiangsu, China) | Cat# C001028 |
|  | | | |
| **Oligonucleotides** | | | |
| Primers for RT-qPCR, see Table 2 | | This paper | N/A |
| UCP1 sgRNA targeting sequence:  TCGGACGTACACCCGACCCT | | GenChem | N/A |
| ANXA1 siRNA | | Ribo Bio | N/A |
| UCP1 siRNA | | Ribo Bio | N/A |
| GATA3 siRNA | | Ribo Bio | N/A |
| Control siRNA | | Ribo Bio | N/A |
|  | |  |  |
| **Recombinant DNA** | | | |
| pGV358-ANXA1 | | This paper | N/A |
| pGV341-UCP1 | | This paper | N/A |
| pCV572-GATA3 | | This paper | N/A |
| pGV388-UCP1 | | This paper | N/A |
| GV736 Ksp-cadherin-CRLS1 | | This paper | N/A |
|  | |  |  |
| **Software and Algorithms** | | | |
| Zen (blue edition) | | Zeis | https://www.zeiss.com.cn/ |
| ImageJ | | National Institutes of Health | https://imagej.nih.gov/ij/ |
| GraphPad Prism 8.0 | | GraphPad | https://www.graphpad.com/ |
| Adobe Photoshop | | Adobe | https://www.adobe.com/cn |
| Adobe Illustrator | | Adobe | https://www.adobe.com/cn |
| Image-ProPlus 6.0 | | AZoM | https://www.azom.com/ |
| Wave | | Agilent | https://www.agilent.com.cn/ |
