# Supplementary material for: ANXA1 improves mitochondrial homeostasis through uncoupling protein 1 in diabetic nephropathy
Source: J Adv Res. 2025 Nov 22;86:689–708. doi: 10.1016/j.jare.2025.11.039 (PMC13453871; doi:10.1016/j.jare.2025.11.039)
Supplement: Supplementary file 4 [file mmc6.docx]

**Figure 1F**

UCP1 33kDa

tubulin 55kDa

ANXA1 37kDa

**Figure 1H**

UCP1 33kDa

ANXA1 37kDa

tubulin 55kDa

**Figure S1A**

ANXA1 Inupt 37kDa

ANXA1-UCP1 37kDa

UCP1 Inupt 33kDa

UCP11-ANXA11 33kDa

**Figure S1D**

p-GATA3 48kDa

GATA3 48kDa

tubulin 55kDa

**Figure S1E**

**Figure S1F**

GATA3 48kDa

tubulin 55kDa

**Figure 2C**

p-GATA3 48kDa

tubulin 55kDa

GATA3 48kDa

**Figure 2D**

tubulin 55kDa

GATA3 48kDa

p-GATA3 48kDa

**Figure 2F**

**Figure 2I**

tubulin 55kDa

UCP1 33kDa

UCP1 33kDa

tubulin 55kDa

**Figure 2L**

UCP1 33kDa

tubulin 55kDa

GATA3 48kDa

**Figure 3D**

UCP1 33kDa

tubulin 55kDa

**Figure 3K**

MFF 34kDa

DRP1 83kDa

tubulin 55kDa

tubulin 55kDa

CRLS1 35kDa

**Figure 5F**

**Figure 5H**

tubulin 55kDa

DRP1 83kDa

MFF 34kDa

**Figure 5J**

tubulin 55kDa

DRP1 83kDa

MFF 34kDa

**Figure S3C**

**Figure S6B**

UCP1 33kDa

tubulin 55kDa

**Figure S6A**

tubulin 55kDa

UCP1 33kDa

**Figure S2E**

**Figure S2E**

UCP1 33kDa

tubulin 55kDa

**Figure S1G**

tubulin 55kDa

ANXA1 37kDa
